# Dual Regulation of Host TRAIP Post-translation and Nuclear/Plasma Distribution by Porcine Reproductive and Respiratory Syndrome Virus Non-structural Protein 1α Promotes Viral Proliferation

**DOI:** 10.3389/fimmu.2018.03023

**Published:** 2018-12-18

**Authors:** Peidian Shi, Yanxin Su, Ruiqiao Li, Lei Zhang, Chen Chen, Lilin Zhang, Kay Faaberg, Jinhai Huang

**Affiliations:** ^1^School of Life Sciences, Tianjin University, Tianjin, China; ^2^Agricultural Research Service, USDA, National Animal Disease Center, Ames, IA, United States

**Keywords:** PRRSV, TRAIP, nsp1α, SUMOylation, IFN, TBK1

## Abstract

In this study, we show that porcine reproductive and respiratory syndrome virus (PRRSV) non-structural protein 1α (nsp1α) facilitates PRRSV escape from innate immune by modulating nuclear to cytoplasmic translocation and distribution ratio of TRAIP to promote virus proliferation. Mechanistically, TRAIP interacts with PRRSV nsp1α via its K205 site, while NSP1α decreases the SUMOylation and K48 ubiquitination independent of the TRAIP interaction K205 site. Modulation of the dual modification of TRAIP by PRRSV nsp1α results in over-enrichment of TRAIP in the cytoplasm. Enrichment of nsp1α-induced cytoplasmic TRAIP in turn leads to excessive K48 ubiquitination and degradation of serine/threonine-protein kinase (TBK1), thereby antagonizing TBK1-IRF3-IFN signaling. This study proposes a novel mechanism by which PRRSV utilizes host proteins to regulate innate immunity. Findings from this study provides novel perspective to advance our understanding in the pathogenesis of PRRSV.

## Introduction

The host innate immune response, predominantly IFN-α and IFN-β, is the first line of defense against pathogens (1, 2). As multi-functional antiviral cytokines, type I interferons can be induced by virus infection (3). The viral RNA is recognized by cytosolic sensors, retinoic acid-inducible gene I (RIG-I) and melanoma differentiation-associated gene 5 protein (MDA5) (4). RIG-I and MDA-5 bind to the mitochondrial adapter protein MAVS/IPS-1, resulting in the activation of IκB kinase-ε (IKKε) or downstream signaling of TRAF family member-associated NF-kappa-B activator (TANK)-binding kinase 1 (TBK1), then phosphorylation of IRF-3, its entry nucleus and final induce IFN-β transcription (5, 6). However, porcine reproductive and respiratory syndrome virus (PRRSV) has developed a variety of strategies to avoid or suppress the host immune response, in particular the interferon (IFN)-mediated innate immune response.

PRRSV, the causative agent of PRRS, is a positive-strand RNA virus that belongs to the family *Arteriviridae* within the order *Nidovirales* (7). PRRSV infection often causes acute reproductive failure in sows and dyspnea in piglets and substantial economic losses each year (8). Accumulating evidence has revealed that PRRSV results in persistent infection due partly to inhibition of the host innate immune response (9–11). The genome of PRRSV is approximately 15 kb and encodes 9 overlapping open reading frames (ORFs) encoding at least 8 structural proteins and 16 non-structural proteins (12, 13). PRRSV non-structural protein 1 (nsp1) contains two papain-like cysteine proteases, papain-like cysteine protease α (PCPα) and papain-like cysteine protease β (PCPβ), and self-cleaves into nsp1α and nsp1β subunits (14, 15). The nsp1α subunit is composed of three distinct functional motifs; a papain-like cysteine protease α motif (PCPα), a N-terminal zinc finger motif (ZF1), and a newly reported C-terminal zinc finger motif (ZF2) (16). PRRSV nsp1α has been reported to inhibit NF-κB activation by targeting linear ubiquitin chain complex (17) and block the transcription of type I interferon by the degradation of CREB-binding protein (CBP) (18). It has also been shown to contribute to PRRSV proliferation, while siRNAs which specifically target nsp1α significantly inhibit the replication of PRRSV in MARC-145 cells (19).

Protein post-translational modifications (PTMs) such as ubiquitination and SUMOylation play a key role in signal transduction pathways in cells (20, 21). The ubiquitination system utilizes the combination of ubiquitin molecules and their target proteins to form polyubiquitin chains (22–24). E3 ubiquitin ligase is a protein acting in the ubiquitination of a particular target protein (25). More recently it has been shown that the ubiquitin proteasome system can regulate the biological functions of tumor cells such as proliferation and metastasis by mediating the degradation of many tumor-related proteins (26, 27).

SUMO is a newly discovered ubiquitin-like molecule and can covalently conjugate proteins throughout the cell (28). At least three highly conserved SUMO proteins (SUMO1/2/3) exist in higher eukaryotic cells, and there is a high degree of homology between SUMO2 and SUMO3 (29). SUMOylation is a dynamic and reversible process catalyzed by SUMO-specific enzyme E1, E2, and E3 (30). The SUMO molecule is covalently linked to the substrate protein, regulating the target protein localization and the interaction of SUMO modified-proteins with their binding partners (31). Consequently, these changes in turn affect signaling mechanisms, which have been shown to regulate many cellular functions such as cell growth, proliferation, apoptosis, DNA repair, and cell survival (32–34).

TRAF-interacting protein (TRAIP), also known as RNF206 (RING-finger protein 206), was initially identified through its ability to bind TRAF1 and TRAF2 in yeast two-hybrid screening (35). TRAIP is indispensable, as mouse embryos fail to develop when TRAIP is knocked out in both mice and *Drosophila* (36, 37). TRAIP has been identified as an E3 ubiquitin ligase and a substrate of SUMOylation, which plays an important biological function. TRAIP is indispensable in the immune response, which negatively regulates TRAF2, tumor necrosis factor receptor 2 (TNFR2), CD30, and TNF mediated NF-κB activation (35, 38). As an E3 ubiquitin ligase, TRAIP directly binds to TBK1, which promotes TBK1 degradation via K48-linked ubiquitination in 293T cells (39).

In our research, the TRAIP gene was cloned from porcine peripheral blood mononuclear cells (PBMCs). The relationship between TRAIP and PRRSV replication was investigated. We verified that TRAIP contributed to the proliferation of PRRSV. The PCPα domain of nsp1α interacts with TRAIP. Interestingly, SUMOylation and self-ubiquitination of TRAIP was attenuated by PRRSV nsp1α. Changes in the dual modification of TRAIP affect its own proportional distribution in the nucleus and the cytoplasm. Functionally, the retention of TRAIP in the cytoplasm facilitates the ubiquitination of TBK1, resulting in the degradation of TBK1, thereby inhibiting the production of type I interferon. Therefore, our study revealed a new model of interaction between viral proteins and cellular hosts, one which aims to suppress type I interferon production, in order to promote PRRSV proliferation. As a crucial mechanism of PRRSV, achieving persistent infection and immunosuppression through nsp1α regulation of nuclear/plasma distribution and modification of TRAIP, our results provide a novel target pathway to develop antivirals against PRRSV.

## Materials and Methods

### Ethics Statement

The protocol was reviewed and approved by the Tianjin University Institutional Animal Care and Use Committee (TJIACUC) (Protocol number: SYXK-Jin 2014-0004). All animal experiments were performed using BALB/c mice and maintained in individually ventilated cages at the Tianjin Laboratory Animals Center. This study was carried out in strict accordance with the recommendations in the Guide for the Care and Use of Laboratory Animals of the Tianjin government authority for the use of animals in experiments.

### Cells, Virus, and Antibody

Porcine peripheral blood mononuclear cells (PBMCs) were isolated from pigs of the Tianjin Ninghe farm according to a previously described protocol (40). PRRSV-permissive PAM cell lines CRL2843-CD163 (3D4/21) and monoclonal antibodies against PRRSV nsp2 were kindly contributed by China Agricultural University. 3D4/21 cells were cultured in RPMI-1640 medium (Gibco, USA) supplemented with 10% (V/V) fetal bovine serum (FBS, Biological Industries) and antibiotic-antimycotic solution. Human embryonic kidney (HEK) 293T cells and HeLa cells were maintained in Dulbecco's modified Eagle's medium (DMEM, Gibco) with 10% FBS supplemented with an antibiotic-antimycotic mixture of 100 mg/ml streptomycin, 100 IU/ml penicillin and 50 U/ml amphotericin B. The cells were maintained in a humidified 5% CO_2_ incubator at 37°C.The PRRSV-JXwn06 strain was used in our study and the titer was determined to be 104 PFU/ml as previously described (41).

Polyclonal antibody against TRAIP was prepared by immunizing BALB/c mice with recombinant His-TRAIP couple with mineral oil adjuvant as previously described (42). Monoclonal antibodies against PRRSV nsp1α and N protein were the gift of Prof. Shaobo Xiao of Huazhong Agricultural University and Jun Han of China Agricultural University, respectively. Labeled antibodies used in the experiments were purchased from Cell Signaling Technology (CST, Danvers, MA, USA) and Applied Biological Materials Inc (ABM, Vancouver, Canada). An internal reference antibody and secondary antibodies were purchased from Invitrogen (Thermo Fisher Scientific, Waltham, MA, USA). Antibodies to β-actin and Histone H3 were purchased from TransGen (Beijing, China) and Santa Cruz Biotechnology (Santa Cruz, CA), respectively.

### Cloning of the Complete Porcine TRAIP CDS

Total RNAs were extracted from PBMC cells using TRIzol reagent (TaKaRa, China). First-strand cDNA synthesis was carried out using reverse transcriptase (TaKaRa). TRAIP was synthesized using the specific primers based on the predicted TRAIP sequence (GenBank Accession Nos. XM_021068793.1) as shown in Table 1, and the amplified fragments were cloned into pGEM^®^-T Easy Vector (Transgen, Beijing).

**Table 1:** Primers used for PCR amplification.

**Primer name**	**Genbank number**	**Sequence of primer(5^′^-3^′^)**
T-TRAIP-F	XM_021068793.1	GAGACCAGTCATGCCTATTCG
T-TRAIP-R		TGTTTTCACTAGGACAGGAAAC
pCMV-TRAIP-F	XM_021068793.1	ATTCGAATTTAAATCGGATCCATGCCTATTCGTGCTCTG
pCMV-TRAIP-R		ATCCTTCGCGGCCGCGGATCCCTAGGACAGGAAACTGT
pet-28a-TRAIP-F	XM_021068793.1	ACAGCAAATGGGTCGCGGATCCATGTATCGTGCAGCGGAT
pet-28a-TRAIP-R		GACGGAGCTCGAATTCGGATCCCAGACTTGTCTCCTCA
pCMV-TRAIP(1-177)-F	XM_021068793.1	CCAGTCGACTCTAGAGGATCCATGCCTATTCGTGCTCTGTGC
pCMV-TRAIP(1-177)-R		CAGGGATGCCACCCGGGATCCCTACCGCTGGCTCTGGAGTAGG
pCMV-TRAIP(1-395)-F	XM_021068793.1	CCAGTCGACTCTAGAGGATCCATGCCTATTCGTGCTCTGTGC
pCMV-TRAIP(1-395)-R		CAGGGATGCCACCCGGGATCCCTACCGGATGAAAACAGGGAAG
pCMV-TRAIP(54-472)-F	XM_021068793.1	ATTCGAATTTAAATCGGATCCATGCAAAAGAACCATTATCA
pCMV-TRAIP(54-472)-R		ATCCTTCGCGGCCGCGGATCCCTAGGACAGGAAACTGTCCAGC
pCMV-TRAIP(178-472)-F	XM_021068793.1	CCAGTCGACTCTAGAGGATCCATGCCTGAGGTGGAGGAAATGAT
pCMV-TRAIP(178-472)-R		CAGGGATGCCACCCGGGATCCCTAGGACAGGAAACTGTCCAGCT
pEGFP-TRAIP-F	XM_021068793.1	GTACCGCGGGCCCGGGATCCATGCCTATTCGTGCTCTG
pEGFP-TRAIP-R		TTATCTAGATCCGGTGGATCCGGACAGGAAACTGTCC
pCMV-nsp1-F	MF187956.1	CCAGTCGACTCTAGAGGATCCATGTCTGGGATACTTGATCGGTG
pCMV-nsp1- R		CAGGGATGCCACCCGGGATCCACCGTACCACTTATGACTGCCAA
pCMV-nsp1α-F	MF187956.1	CCAGTCGACTCTAGAGGATCCATGTCTGGGATACTTGATCGGTG
pCMV-nsp1α-R		CAGGGATGCCACCCGGGATCCCTACATAGCACACTCAAAAGGGC
pCMV-nsp1β-F	MF187956.1	CCAGTCGACTCTAGAGGATCCATGGCTGACGTCTATGACATTGGT
pCMV-nsp1β-R		CAGGGATGCCACCCGGGATCCCTAACCGTACCACTTATGACTGC
pCMV-nsp1α N(1-167)-F	MF187956.1	CCAGTCGACTCTAGAGGATCCATGTCTGGGATACTTGATCGGTG
pCMV-nsp1α N(1-167)-R		CAGGGATGCCACCCGGGATCCCTACCTCTGCGGGAGCGGCAA
pCMV-nsp1α N(67-180)-F	MF187956.1	CCAGTCGACTCTAGAGGATCCATGACTGTCGAGTGCTCCCCCG
pCMV-nsp1α N(67-180)-R		CAGGGATGCCACCCGGGATCCCTACATAGCACACTCAAAAGGGC

### Plasmid Construction

The pFLAG-CMV2-TRAIP, pMyc-CMV2-TRAIP, pHA-CMV2-TRAIP, and pEGFP-TRAIP eukaryotic expression vector was constructed, respectively. The specific primers pairs (Table 1), harboring common sequence with the vector, were used to amplify the TRAIP gene and ligated with pFlag-CMV2, pMyc-CMV2, pHA-CMV2, and pEGFP vector, respectively by using a one-step clong kit (Vazyme, Nanjing, China). The prokaryotic expression plasmid pet-28a-TRAIP was constructed using primers pet-28a-TRAIP-F and pet-28a-TRAIP-R (Table 1), to express the recombinant protein His-TRAIP.

### Transcriptome Sequencing and Analysis

3D4/21 cells were grown on 6-well plates until the cell density was about 70~80%, and then were inoculated 0.5 MOI PRRSV for 24 h. Virus-infected cells were washed twice with cold PBS and added 1 ml Trizol. The treated cells were sent to the Guangzhou GENE DENOVO Company for transcriptional sequencing. The obtained transcriptome data and a heatmap of differentially expressed genes was analyzed using an online website (https://software.broadinstitute.org/morpheus/).

### Quantitative Reverse Transcription PCR (RT-qPCR)

First-strand cDNA was synthesized from purified RNAs of 3D4/21 cells or HEK293T cells using a First-Strand Synthesis System (Transgen, Beijing, China) according to the manufacturer's instructions. The relative gene expression was analyzed by qRT-PCR that was performed on an ABI 7500 Real-time PCR system (Applied Biosystems, Foster City, CA, USA). The comparative cycle threshold (CT) method was used to calculate the relative gene expression levels according to manufacturer's protocol (Applied Biosystem). All data presented was relatively quantitative, based on the mRNA level of the endogenous gene β-actin and analyzed using GraphPad Prism 6.0 software. All of the primers pairs used for quantitative real-time PCR are listed in Table 2.

**Table 2:** Primers used for qRT-PCR amplification.

**Primer name**	**Genbank number**	**Sequence of primer(5^′^-3^′^)**
PRRSV-N-F	KX286735.1	GCCTCGTGTTGGGTGGCAGA
PRRSV-N-R		CACGGTCGCCCTAATTGAATAGG
TRAIP-F	XM_021068793.1	GGAAGCACATTCTCCCGTTCA
TRAIP-R		GGCGGATCATAGTCGTGTCAGTA
TNF-α-F	X57321	GAGATCAACCTGCCCGACT
TNF-α-R		CTTTCTAAACCAGAAGGACGTG
IFN-β-F	NM_001003923	GCAGTATTGATTATCCACGAGA
IFN-β-R		TCTGCCCATCAAGTTCCAC
NF-κB-F	X61498.1	CCCAGCCATTTGCACACCTCAC
NF-κB-R		TTCAGAATTGCCCGACCAGTTTTT
β-actin-F	DQ452569.1	GAATCCTGCGGCATCCACGA
β-actin-R		CTCGTCGTACTCCTGCTTGCT

### Confocal Immunofluorescence

The procedure for confocal microscopy has been described previously (41). HeLa cells or 3D4/21 cells were seeded on 12-well plates until the cell density was about 30~40%. Depending on the specific experiment, the cells were transfected with plasmid expressing TRAIP and/or nsp1α with different labels or empty vector (pFlag-CMV2, pMyc-CMV2, pHA-CMV2, or pEGFP vector) (0.5 μg). In order to detect endogenous immunofluorescence, 3D4/21 cells were infected with 0.5 MOI of PRRSV and incubated at 37°C. At 18 h post-transfection or infection, the cells were fixed with 4% paraformaldehyde for 15 min and then permeabilized with PBS containing 0.3% Triton X-100 for 10 min at room temperature. Then cells were blocked for 30 min with 1% bovine serum albumin (BSA) and incubated with primary antibodies (anti-Myc, anti-HA, anti-Flag) or anti-PRRSV nsp1α antibody (diluted at 1:200) at room temperature (RT) in a humid chamber followed by 10 min washing in PBS. Secondary antibodies (FITC-conjugated anti-mouse IgG or PE-conjugated anti-rabbit IgG) (diluted at 1: 200) were used and nuclear DNA was stained with 4′,6-diamidino-2-phenylindole (DAPI). Finally, the localization of TRAIP and nsp1α or TBK1 was observed with an Olympus confocal microscope. Images were taken at × 100 magnification.

### Luciferase Assay

293T cells were seeded into 24-well plates and transfected with the TRAIP and/or nsp1α expression vectors, along with a luciferase reporter (IFNβ-Luc or ISRE-Luc) and the internal control LacZ. At 12 h post-transfection, the cells were infected with Sendai virus (SeV) at a MOI of 0.5. The lysed samples were prepared, and the luciferase activity was measured using the multimode microplate reader (Promega) according to the manufacturer's recommendations.

### Western Blot Analyses

Transfected or virus-infected cells were washed twice with cold PBS and lyzed in RIPA buffer (Solarbio, Beijing, China) containing the proteinase inhibitors 20 nM phenylmethanesulfonyl fluoride [PMSF] or 2.5 mM desumoylation protease inhibitor N-Ethylmaleimide [NEM] (Sigma, USA).

Cell lysates were boiled in buffer for 10 min and separated with 12% SDS-PAGE. The separated proteins were transferred to the methanol-activated PVDF membrane (Millipore). Membranes were blocked with 5% non-fat dry milk in TBST (0.05% Tween-20) for 1 h and incubated with an antibody against PRRSV nsp1α (1:2,000), PRRSV N (1:5,000), TRAIP (1:500), β-actin (1:5,000) or labeled antibodies (1:5,000) for overnight at 4°C, followed by washing and incubation with HRP-conjugated antibody for 1 h at room temperature. Immunodetection was completed using Pierce ECL Western Bloting Substrate (Thermo Scientific).

### Immunoprecipitation

For co-immunoprecipitation, cells were cultivated in 60 mm plates and transfected with Flag-TRAIP, Myc-nsp1α, or related expression plasmid. At 24 h post-transfection, the cells were lysed in 500 μL RIPA lysis buffer supplemented with protease inhibitor PMSF or NEM. The cell lysates were incubated with anti-Myc or anti-Flag labeled beads (Sigma, St. Louis, MO, USA) for 2 h or overnight at 4°C, followed by washing three times with lysis buffer for 10 min each and boiled for 5 min with protein loading buffer. Proteins bound to the beads were separated by SDS-PAGE and western blotting was performed as described above.

### Flow Cytometry Analysis

3D4/21 cells were infected with PRRSV or transfected with the TRAIP plasmid. The cells were harvested at the indicated times, followed by washing with PBS twice and digested with trypsin. Pre-cooled 80% ethanol was added to the harvested cells for 1 h. Cells were washed with PBS twice and stained with anti-TRAIP antibody or anti-PRRSV nsp2, and incubated with goat anti-mouse IgG FITC conjugate (1:200) for 30 min. Fluorescence-activated cell sorting was performed on a FACS LSR II (BD Biosciences, San Jose, CA, USA). A total of 1 × 10^5^ cells was analyzed per run.

### RNA Interference

A small RNA interfering (siRNA) assay was performed to confirm the target gene of TRAIP (siTRAIP) and a negative control RNA (NC), synthesized by GenePharma (Shanghai, China) (Table 3). Briefly, 3D4/21 cells were seeded in 12-well plates (60–80% confluence) and transfected with siRNA1 (siTRAIP-1) or siRNA2 (siTRAIP-2) at a final concentration of 50 nmol/L using Lipofectamine3000 (Invitrogen). The cells were infected with 0.5 MOI PRRSV and harvested after 24 h. The gene expression levels were confirmed by quantitative real-time PCR (qRT-PCR) and western blotting.

**Table 3:** Primers used in the small RNA interfering assay.

**Primer name**	**Primer sequence (5^′^-3^′^)**
Negative control	F:UUCUCCGAACGUGUCACGUTT
	R:ACGUGACACGUUCGGAGAATT
siTRAIP-1	F:GCACUAUAUGCUCCGACUUTT
	R:AAGUCGGAGCAUAUAGUGCTT
siTRAIP-2	F:GGAGGAGAGUGUCUUAGAUTT
	R:AUCUAAGACACUCUCCUCCTT

### Preparation of Nuclear and Cytoplasmic Extracts

HEK 293T cells were co-transfected with Myc-nsp1α and Flag-TRAIP (WT) or Flag-TRAIP (K205R) plasmids in the presence of the proteasome inhibitor MG132. Cytoplasmic and nuclear proteins were extracted using a Nuclear and Cytoplasmic Protein Extraction Kit (Beyotime Institute of Biotechnology, China) according to the manufacturer's instructions. Briefly, the treated cells were washed with PBS and collected. The cell pellet was completely suspended and dispersed with 200 μl cytoplasmic protein extraction reagent A. Next, 10 μl cytoplasmic protein extraction reagent B was added to the cell suspension on ice for 15 min. Cytoplasmic proteins are collected after centrifugation. Resuspend the nuclear pellet in 50 μl of ice cold nuclear extraction buffer. The nuclear fraction was collected after shaking and centrifugation. Abundance of TRAIP in the nucleus and cytoplasmic was detected by western blotting.

### Statistical Analysis

Data were subjected to one-way analysis of variance (one-way ANOVA) and expressed as mean ± SEM. Pairwise multiple comparison was conducted to determine which group differed by two-way ANOVA followed by Bonferroni post-tests using Prism 6.0 (GraphPad Software Inc.). Results were considered statistically significant if *P* < 0.05.

## Results

### Up-Regulation of TRAIP Accumulation in 3D4/21 by PRRSV

**Figure 1:**
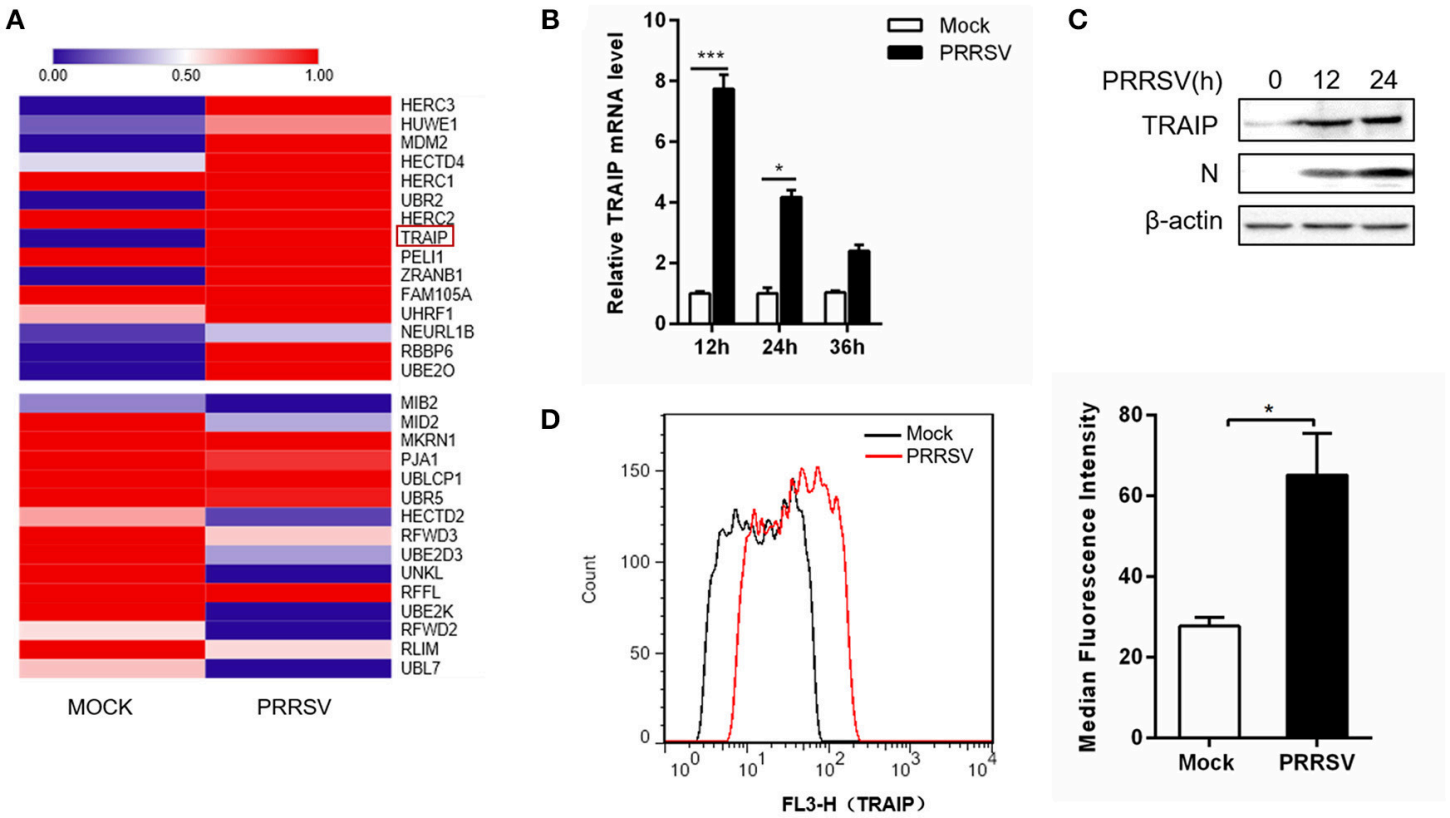
TRAIP is upregulated in 3D4/21 by PRRSV. **(A)** The heatmap of ubiquitin-related differentially expressed genes analyses were generated using online websites (https://software.broadinstitute.org/morpheus/). Red bars indicate an upregulation in expression of at least 1x fold. **(B)** qRT-PCR analysis of TRAIP in 3D4/21 inoculated without or with 0.5 MOI PRRSV at the indicated times. **(C)** 3D4/21 were either mock-infected or infected with PRRSV at a 0.5 MOI for 12 or 24 h. The cells were then harvested to detect TRAIP by Western blotting using a mouse anti-TRAIP polyclonal antibody and cell lysates were then analyzed for expression of PRRSV structural protein (N) with anti-N MAb. **(D)** 3D4/21 cells were mock-infected or infected with 0.5 MOI PRRSV. Cells were collected at 12 h post-infection. Analysis of TRAIP protein expression levels by flow cytometry. ^*^P < 0.05, ^***^P < 0.001 (analysis of two-way ANOVA followed by Bonferroni post-test). Data are representative of three independent experiments.

The RNA expression profiles of PRRSV-infected 3D4/21 were performed by high throughput RNA sequencing (RNA-Seq). A transcriptome analysis from 3D4/21 cells was performed to search for genes related to antiviral immunity that are significantly altered after PRRSV infection. The mRNA expression profiles of the E3 ubiquitin ligase family revealed an up-regulation of TRAIP in PRRSV-infected PAM cells (Figure 1A). To validate the results of the RNA-Seq data mining, 3D4/21 cells were inoculated with 0.5 MOI PRRSV for the indicated times. The dynamic expression of TRAIP in PRRSV-infected 3D4/21 cells was detected by qRT-PCR. The results showed that the mRNA level of TRAIP increased after PRRSV infection, especially at 12 h (Figure 1B). A similar increase of TRAIP protein in infected 3D4/21 cells was confirmed by western blotting (Figure 1C) and flow cytometry (Figure 1D). These data indicated that PRRSV infection led to an up-regulation of TRAIP. The changes in TRAIP expression levels suggests its potential involvement in the proliferation of PRRSV.

### TRAIP Impact on PRRSV Proliferation

**Figure 2:**
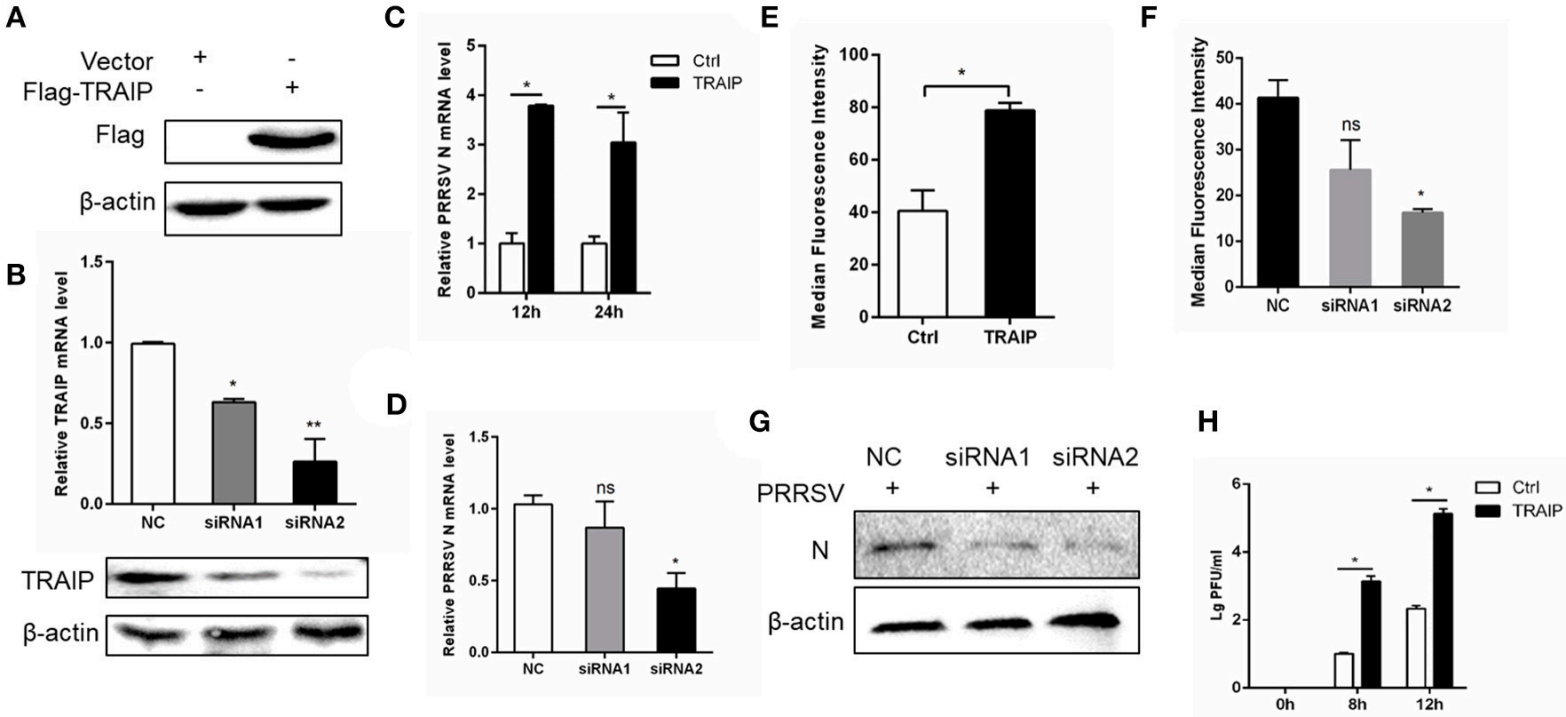
TRAIP contributes to PRRSV proliferation. Flag-tagged TRAIP plasmid or negative control (NC) and siRNA1 (siTRAIP-1) or siRNA2 (siTRAIP-2) targeting TRAIP were transfected into 3D4/21 respectively at the indicated times **(A–D)**, after which the cells were infected with 0.5 MOI PRRSV and the cells were collected at 12 or 24 h post-infection. Flag-TRAIP was recognized by the anti-FLAG tag antibody **(A)** and siRNA interference effects were detected by TRAIP polyclonal immunoblotting and qRT-PCR at post-transfection 24 h **(B)**. The infected cells were collected at 12 h **(C–H)** or 24 h **(C)** post-infection, PRRSV N mRNA was detected by qRT-PCR, and PRRSV N was shown by WB analysis **(G)**. Analysis of PRRSV levels by flow cytometry detection of PRRSV N **(E,F)**. **(H)** PRRSV was inoculated after transfection with TRAIP and PRRSV load was tested by TCID50 after 8 or 12 h. ^*^P < 0.05, ^**^P < 0.01 (analysis of two-way ANOVA followed by Bonferroni post-test). Data are representative of three independent experiments.

To investigate if the expression of TRAIP has an effect on PRRSV proliferation, a PRRSV-infected 3D4/21 cell model was developed. First, we constructed a eukaryotic expression vector (Flag-TRAIP) and designed siRNA sequences targeting TRAIP (Table 3). Western blot analysis revealed that the eukaryotic expression vector of TRAIP was successfully constructed (Figure 2A) and the expression of TRAIP was dramatically silenced by TRAIP siRNA1 (siTRAIP-1) or siRNA2 (siTRAIP-2), especially with siRNA2 (Figure 2B). Next, we attempted to detect the effects of TRAIP overexpression or interference on PRRSV proliferation. Compared to the control sample, the overexpression of TRAIP corresponded to an increase in PRRSV N gene mRNA (Figure 2C). The mRNA level of PRRSV N gene exhibited a downward trend in TRAIP siRNA transfected cells (Figure 2D). In line with that, the results of flow cytometry analysis further confirmed that TRAIP expression levels were consistent with PRRSV proliferation (Figures 2E,F). The results of western blotting also confirmed these findings (Figure 2G). Meanwhile, the virus titer was significantly higher in TRAIP plasmid transfected cells compared to the control (Figure 2H). These data indicate that the changes of cellular TRAIP is consistent with PRRSV proliferation, and TRAIP expression promotes PRRSV proliferation.

### TRAIP Interacts With Nsp1α

We have found that changes in TRAIP expression levels induced by PRRSV infection appeared to be associated with early stage of PRRSV infection. Besides, TRAIP has been reported to be a gene involved in innate immunity (39). Therefore, we further explored whether there is an interaction between the TRAIP and PRRSV non-structural proteins nsp1, nsp4, nsp11, each of which is a major protein involved in inhibiting the IFN-β and NF-κB promoters (41, 43, 44). We first observed that Flag-tagged TRAIP interacted with Myc-nsp1 (Supplementary Figure 1), and the interaction between TRAIP and nsp1 was confirmed by co-immunoprecipitation (Supplementary Figure 1a). Immunofluorescence of both Myc-nsp1 and Flag-TRAIP showed that TRAIP and nsp1 co-localized around the nucleus at 18 h post-transfection (Supplementary Figure 1b). At the same time, we found that the TRAIP protein could also be localized to the cytoplasm as well as the nucleus. TRAIP has been shown to have the ability to shuttle between the nucleus and cytoplasm (39, 45). GFP labeled TRAIP protein was distributed in the cytoplasm as well as showing a punctate distribution in the nucleus (Supplementary Figure 2a). A similar distribution pattern of Myc-nsp1α was also observed (Supplementary Figure 2b).

**Figure 3:**
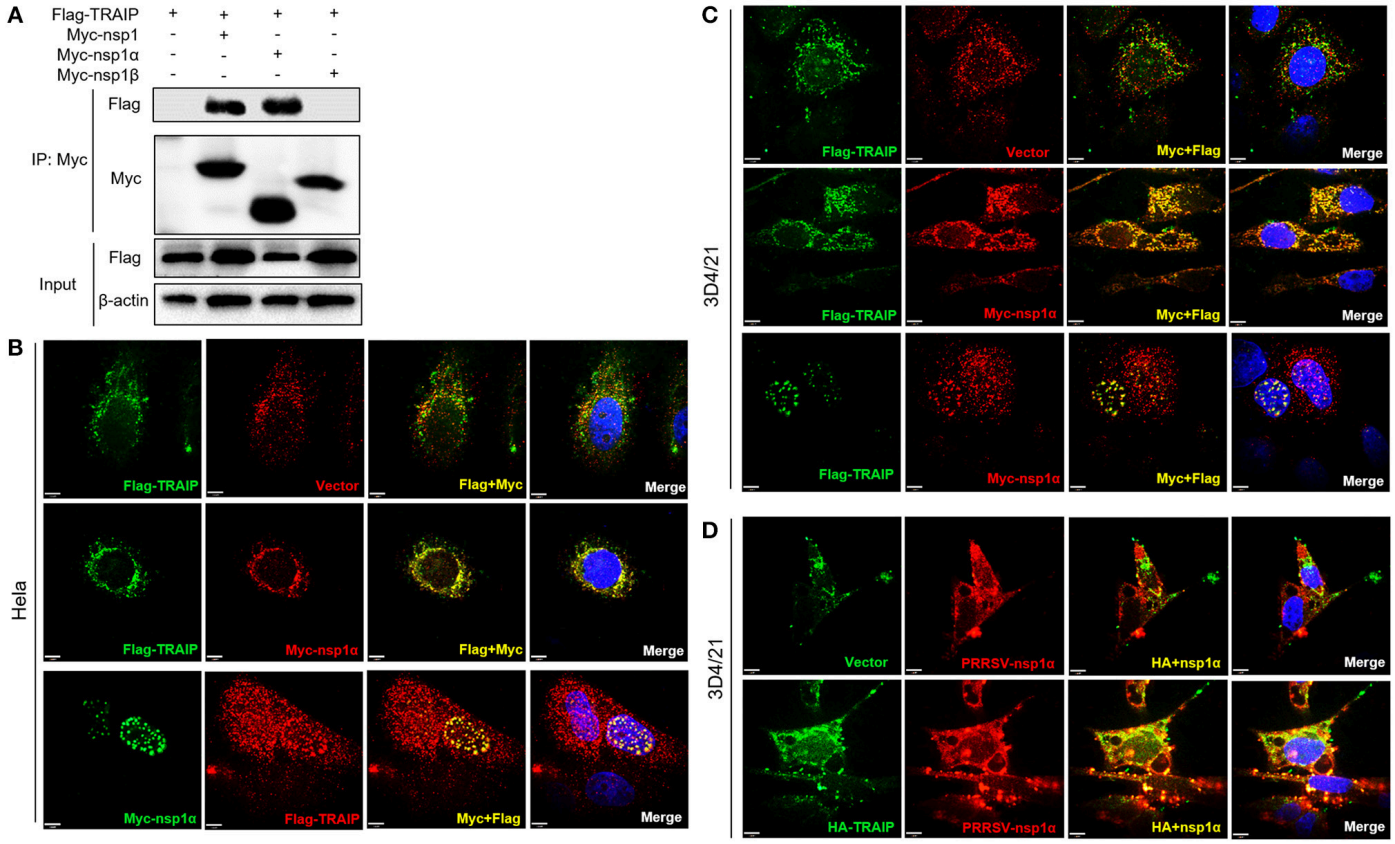
TRAIP interacts with PRRSV-nsp1α. **(A)** HEK293 cells were first transfected with Flag-TRAIP and then co-transfected with Myc-nsp1, Myc-nsp1α, Myc-nsp1β, vector expressing plasmids. The cell lysates were then immunoprecipitated with an anti-Myc MAb and detected by Western blotting at 24 h post-transfection. **(B,C)** Co-localization of nsp1α protein with TRAIP in the cytoplasm and nucleus in HeLa cells and 3D4/21 cells. HeLa or 3D4/21 cells were seeded in 12-well plates and co-transfected with Flag-TRAIP and Myc-nsp1α expressing plasmid. At 18 h post-transfection, the cells were incubated with a rabbit anti-Myc mAb and a mouse anti-Flag antibody followed by FITC-conjugated anti-mouse IgG (green) and PE-conjugated anti-rabbit IgG (red) or FITC-conjugated anti-rabbit IgG (green) and PE-conjugated anti-mouse IgG (red). **(D)** Porcine TRAIP co-localize with PRRSV-nsp1α in PRRSV infection of 3D4/21 cells. Nuclei were stained with DAPI (blue). Cells were observed under a laser confocal imaging analysis system, scale bar: 7 μm.

In PRRSV-infected cells, the nsp1 protein is processed co-translationally into nsp1α and nsp1β. Therefore, we further examined whether nsp1α and/or nsp1β could interact with TRAIP. Co-immunoprecipitation analysis showed that TRAIP specifically interacts with nsp1α, but not nsp1β (Figure 3A). An indirect immunofluorescence assay revealed that Myc-nsp1α and Flag-TRAIP were colocalized both in the cytoplasm and nucleus of HeLa cells (Figure 3B) and 3D4/21 cells (Figure 3C). Similarly, colocalization of HA-TRAIP with nsp1α in PRRSV-infected 3D4/21 cells further confirmed the interaction between TRAIP and nsp1α (Figure 3D). Taken together, our findings indicated TRAIP interacts with nsp1α.

### Nsp1α Removes Sumo-Modification of TRAIP

**Figure 4:**
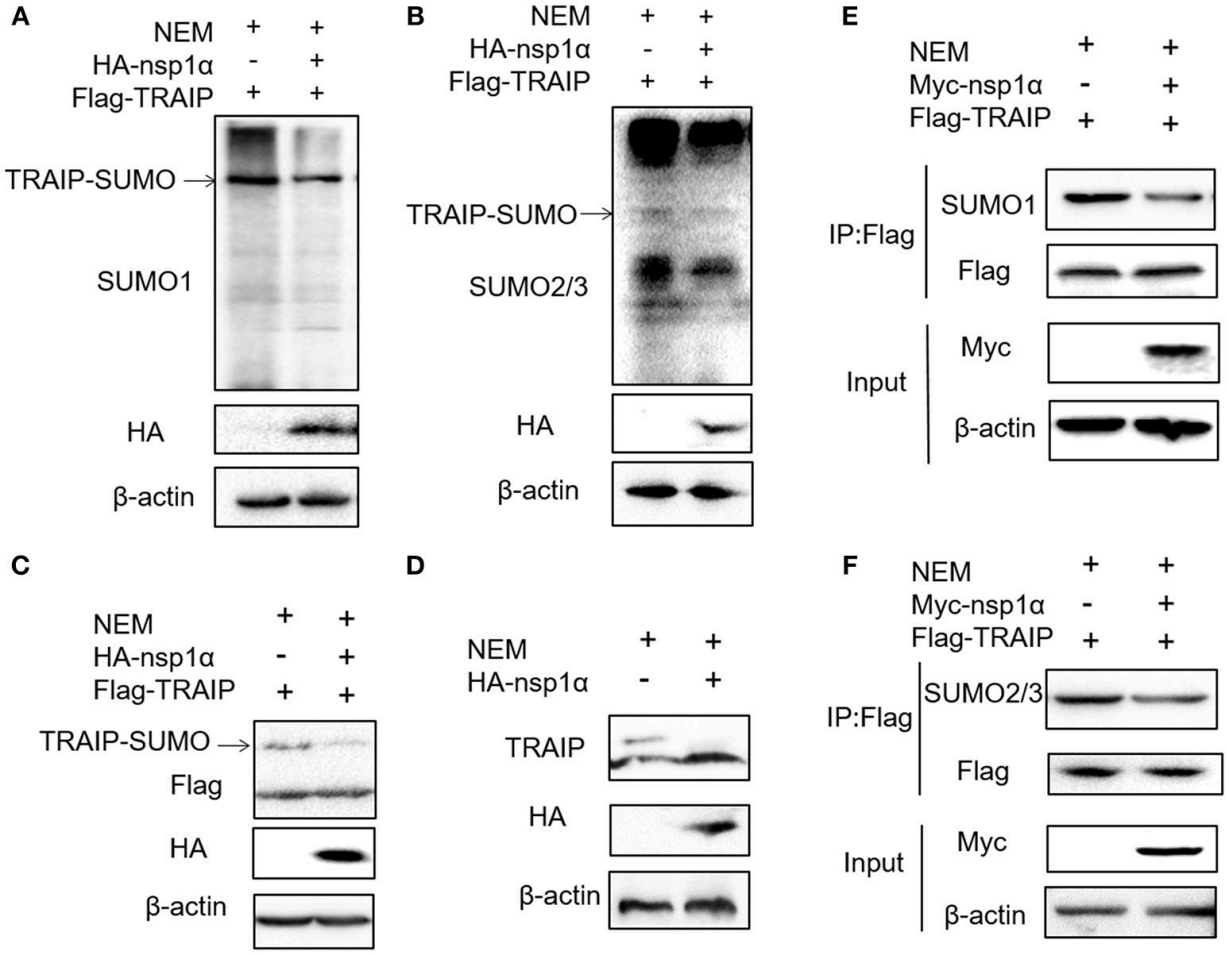
Nsp1α removed SUMO-modification of TRAIP. HEK 293T cells were transfected with HA-nsp1α and Flag-TRAIP, and lyzed in RIPA buffer containing desumoylation protease inhibitor N-Ethylmaleimide [NEM] inhibition of deSUMOylation of TRAIP. The effect of nsp1α on TRAIP SUMO modification was detected using SUMO1 antibody **(A)** or SUMO2/3 antibody **(B)**. **(C)** HEK 293T cells were co-transfected with HA-nsp1α and Flag-TRAIP, and the effect of nsp1α on exogenous TRAIP SUMO-modification was detected by Western blotting. **(D)** HEK 293T cells were transfected with HA-nsp1α or vector and the effect of nsp1α on endogenous TRAIP SUMO modification was detected by WB. HEK 293T cells were cotransfected with Myc-nsp1α and Flag-TRAIP **(E,F)** and the effect of nsp1α on endogenous TRAIP SUMO modification was detected by co-immunoprecipitation.

Previous studies have shown that TRAIP is post-translationally modified by SUMO (46). Some viral proteins can also affect the SUMOylation of host cellular proteins to impact various intracellular activities (47, 48). Overexpression of PRRSV nsp1α weakened the SUMO modification of cells (Supplementary Figure 3). Next, the effect of nsp1α on TRAIP SUMO modification was detected using SUMO1 antibody or SUMO2/3 antibody. We found a pronounced TRAIP sumo-modified band appeared, especially the sumo1 modification, and was attenuated in the TRAIP and nsp1α co-expression groups (Figures 4A,B). At the same time, TRAIP expression is detected by western blotting using anti-Flag antibody and TRAIP antibody, respectively. Our results suggested that Flag-TRAIP or endogenous TRAIP is modified with SUMO moieties. However, SUMOylation of TRAIP is reduced when PPRSV nsp1α is overexpressed (Figures 4C,D). To more accurately detect the effect of nsp1α on sumoylated TRAIP, a co-immunoprecipitation assays was used. The co-immunoprecipitation results further confirmed the effect of nsp1α on the SUMOylation of TRAIP (Figures 4E,F). Taken together, these findings demonstrate that PRRSV nsp1α can significantly reduce the SUMO modification of TRAIP.

### The LZ Domain of TRAIP Interacts With PCPα of Nsp1α

**Figure 5:**
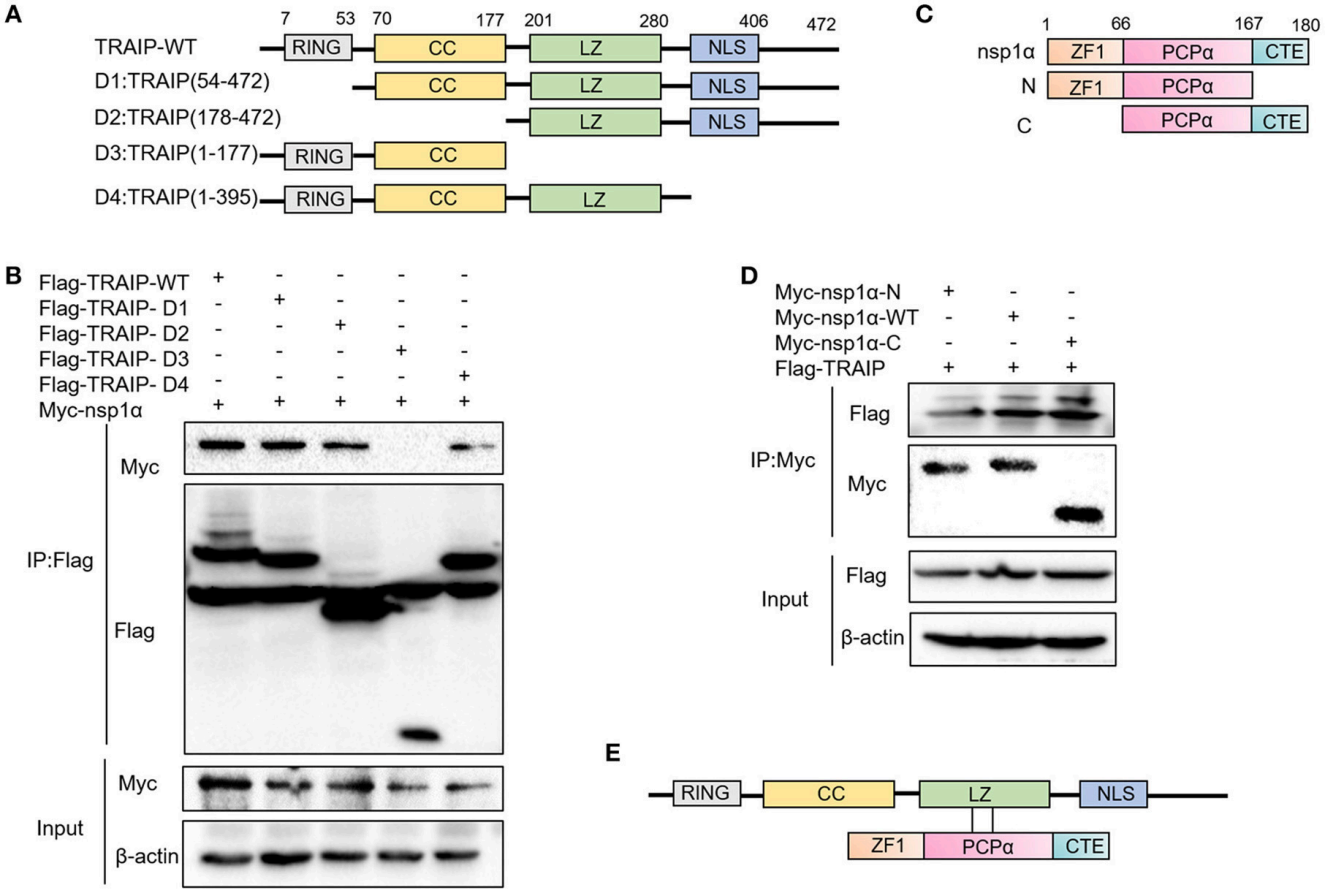
The LZ domain of TRAIP interacts with PCPα of nsp1α. **(A)** Mapping of wild-type (WT) TRAIP and different key enzyme truncated of TRAIP. TRAIP is consisted of a RING domain, a Coiled coil (CC) domain, a Zipper domain, and nuclear localization signals (NLS). **(B)** HEK293T cells were co-transfected with Myc-nsp1α and different Flag-tag TRAIP deletion mutants plasmids. Cells were harvested and lysed, and immunoprecipited (IP) with Flag-conjugated beads. **(C)** Mapping of wild-type (WT) nsp1α and internal deletion mutants. Nsp1α is consisted of a papain-like cysteine protease α (PCPα) motif, an N-terminal zinc finger motif (ZF1), and the carboxyl-terminal extension (CTE). **(D)** Flag-TRAIP interacts with the PCP1α domain of nsp1α. **(E)** The model of TRAIP interacts with nsp1α was proposed.

Previous experiments have verified the interaction of nsp1α with TRAIP. To further provide a molecular mechanism for this interaction, the domain of TRAIP was analyzed and different TRAIP truncations were constructed. Analysis showed that TRAIP consisted of a RING domain, putative coiled-coil domain and leucine zipper region (Figure 5A). The immunoprecipitation results indicated that Flag-TRAIP could be co-precipitated with Myc-nsp1α and the key to this interaction was the LZ domain (residues 201–280) (Figure 5B). Subsequently, to identify the critical domains of nsp1α that were responsible for TRAIP binding, we mapped the domains of PRRSV nsp1α and constructed the corresponding truncated segments, which consisted of amino acids (aa) 1–167 (nsp1α-N), aa 67–180 (nsp1α-C) (Figure 5C). We next investigated the PCPα motif interacted with intact TRAIP (Figure 5D). All nsp1α segments were shown to interact when analyzed by immunoprecipitation. Together these results revealed that the PRRSV nsp1α and TRAIP interaction depended on the PCPα motif of nsp1α and LZ domain of TRAIP (Figure 5E).

### The Effect of PRRSV Nsp1α on TRAIP SUMOylation Is Independent of the Interaction

**Figure 6:**
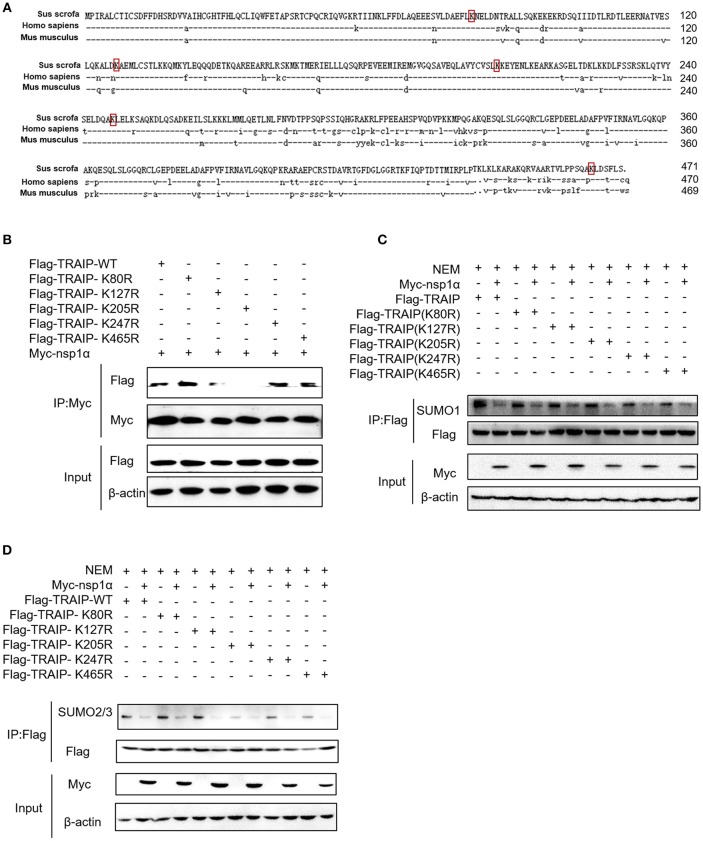
The effect of nsp1α on SUMOylation of TRAIP is independent of their interaction. **(A)** Sequence comparison of TRAIP and homologs from other species. The TRAIP cDNA encoded an ORF with 471 amino acid-long protein, GeneBank Accession XM_021068793.1. Porcine TRAIP amino acid sequence was compared with those from Homo sapiens TRAIP (NM_005879.2), Mus musculus (NM_011634.3), respectively. **(B)** Coimmunoprecipitation analysis of TRAIP K205R SUMO site interacts with Myc-nsp1α in HEK293T cells. **(C,D)** HEK 293T cells were cotransfected with Myc-nsp1α and Flag-TRAIP or different point mutants, and the effect of nsp1α on endogenous TRAIP SUMO modification was detected by co-immunoprecipitation.

Five SUMOylation sites in TRAIP (K80R, K127R, K205R, K247R, and K465R) have been identified in previous studies (46). In this study, the complete CDS of TRAIP was successfully amplified, and amino acid sequence alignment analysis was performed, indicating that TRAIP has high amino acid sequence identity with Homo sapiens and Mus musculus. It is worth noting that the TRAIP's SUMOylation sites are highly conserved (Figure 6A). We constructed TRAIP point mutations to determine whether the SUMOylation site was important for its association with nsp1α. Co-immunoprecipitation analysis indicated that the K205 residue of TRAIP was critical for the interaction (Figure 6B). Next, we sought to investigate the effect of nsp1α on the SUMOylation function of different TRAIP mutants. Surprisingly, nsp1α removed SUMO modifications of different mutants to a similar extent, including K205 (Figures 6C,D). Therefore, nsp1α reduces the SUMO modification of TRAIP independent of the interaction. It was hypothesized that nsp1α may affect the multi-step enzymatic process in SUMOylation or deSUMOylation.

### Nsp1α Inhibits K48-Linked Polyubiquitination and Degradation of TRAIP

Recent analyses suggest an evolutionarily conserved and fundamental molecular interface between the SUMO and ubiquitin systems (49). Protein SUMOylation can influence its subsequent ubiquitination and degradation (50, 51). Studies have shown that TRAIP can be ubiquitinated, and SUMOylation of TRAIP affects its ubiquitination (46). So the next step, the role of nsp1α on TRAIP ubiquitination was investigated.

**Figure 7:**
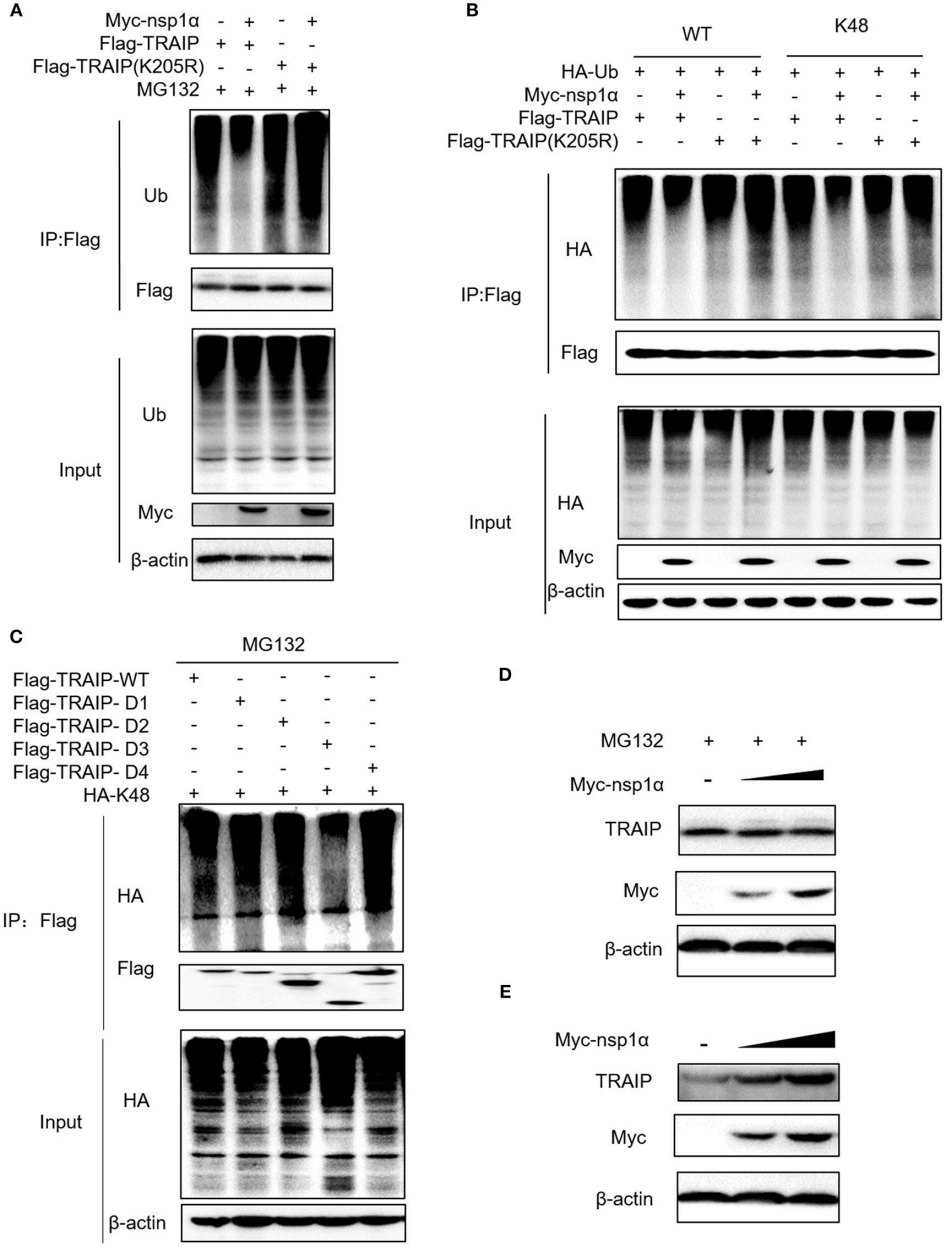
Nsp1α inhibits K48-linked polyubiquitination and degradation of TRAIP. HEK 293T cells were co-transfected with Flag-TRAIP and Myc-nsp1α or vector supplemented 10 μM MG132 **(A)**. At 24 h post-transfection, the cell lysates were co-immunoprecipitated with an anti-Flag and probed with Ub antibody to detect ubiquitin levels of TRAIP respectively by Western blotting. **(B)** 293T cells were co-transfected with Flag-TRAIP or Flag-TRAIP (K205R), HA-Ub-WT, HA-Ub-K48 (ubiquitin mutants retaining a single lysine residue), and Myc-nsp1α or vector. At 24 h post-transfection, the cell lysates were precipitated with an anti-Flag MAb and further detected by Western blotting with an anti-HA MAb and an anti-Flag. **(C)** The K48 polyubiquitination of TRAIP was detected in different TRAIP deletion mutants. HEK 293T cells were transfected with Myc-nsp1α (0.5 or 1.0 μg) or vector **(D,E)**. TRAIP expression in total cellular protein and the protein stability of TRAIP in the absence of proteasome inhibitor MG132 (5 μM) was detected by Western blotting, respectively.

The results revealed that nsp1α inhibited the ubiquitination of TRAIP-WT, but not TRAIP-K205R (Figure 7A). Further analysis found that PRRSV nsp1α inhibited K48-linked polyubiquitination in TRAIP (Figure 7B). As shown in Figure 7B, mutation of lysine 205 to arginine did not affect its ubiquitination level, suggesting that K205 is not a ubiquitination site (lane 7 and lane 5). However, the nsp1α lost the ability to inhibit polyubiquitination of TRAIP-K205R mutant, indicating that nsp1α is dependent on K205 site to reduce the self-ubiquitination of TRAIP. Further analysis showed the K48 polyubiquitination of TRAIP was inhibited by deleting the LZ domain of TRAIP, indicating the presence of the K48 ubiquitination site in the LZ domain (Figure 7C). Next, changes in protein levels caused by nsp1α-induced ubiquitination regulation of TRAIP were verified. When the proteasome degradation of TRAIP was inhibited by MG132, the intracellular TRAIP protein content was not affected in overexpressed nsp1α cells, indicating that nsp1α does not affect the production of TRAIP protein (Figure 7D). However, nsp1α co-transfection can increase the protein stability of TRAIP in the absence of MG132 (Figure 7E). Taken together, nsp1α co-transfection can inhibit K48-linked self-ubiquitination of TRAIP possibly occupy or occlude the ubiquitination site in the LZ domain by changing the TRAIP spatial conformation, thereby inhibiting the degradation of TRAIP and maintaining TRAIP stability.

### Nsp1α Decreased the Abundance of TRAIP in the Nucleus

**Figure 8:**
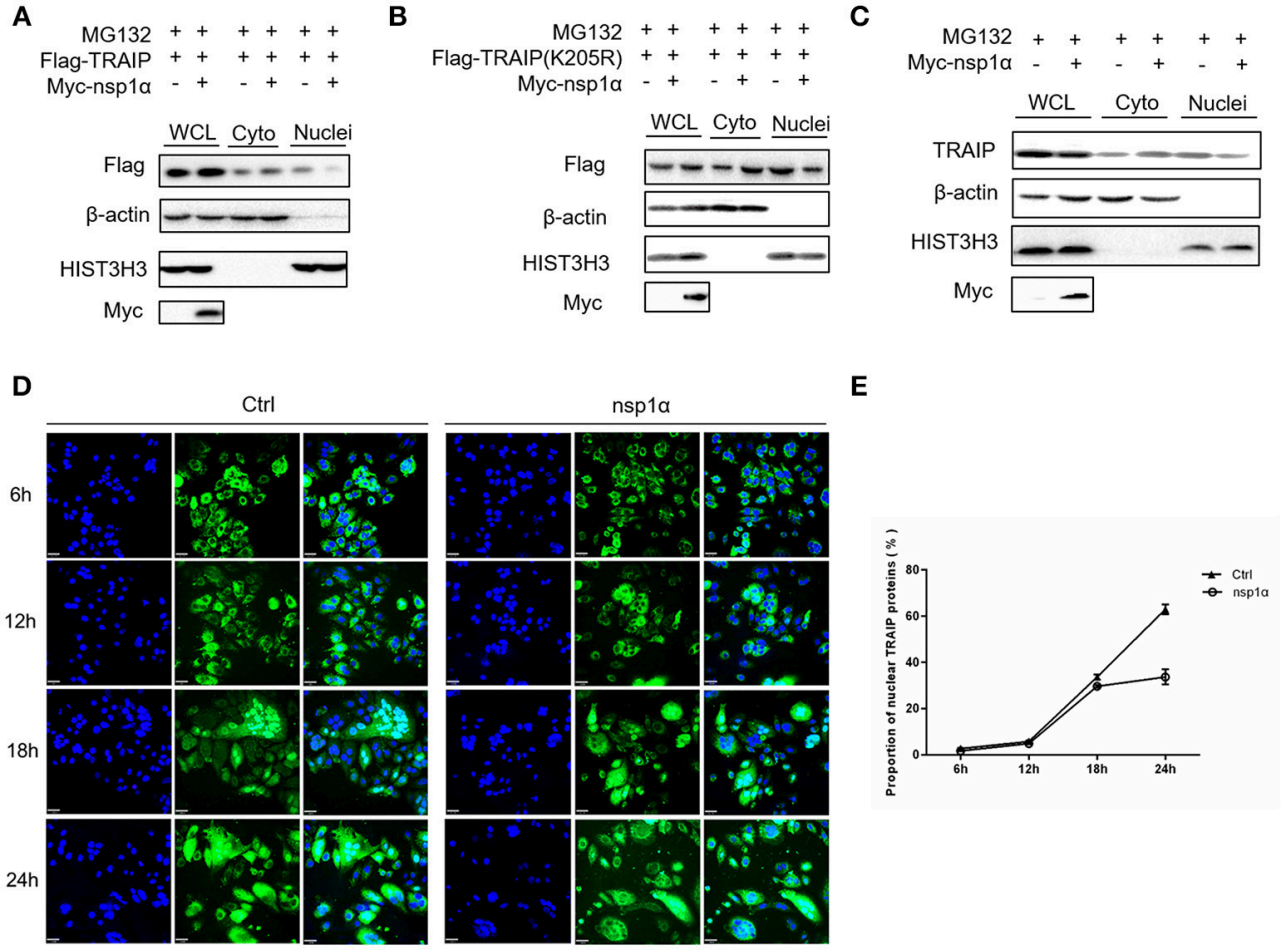
Nsp1α decreased the abundance of TRAIP in the nucleus. HEK 293T cells were co-transfected with Myc-nsp1α and Flag-TRAIP (WT) **(A)** or Flag-TRAIP (K205R) **(B)**, in the presence of proteasome inhibitor MG132 (5 μM) and after 24 h, cytoplasmic protein and nucleoprotein were extracted and detected with anti-Flag mAb or anti-Myc antibody, respectively, by Western blotting. HIST3H3 was used as an internal loading control for nuclear protein load, and β-actin as the control for cytoplasmic protein load. **(C)** HEK 293T cells were transfected with Myc-nsp1α. At 24 h post-transfection, cytoplasmic protein and nucleoprotein were extracted and detected with TRAIP mAb or anti-Myc antibody. **(D)** Co-transfection of Flag-TRAIP with Myc-nsp1α or vector into HeLa cells. The cells were fixed and double-stained with a mouse anti-Flag antibody at the indicated times, followed by FITC-conjugated anti-mouse IgG (green). Nuclei were stained with DAPI (blue). Cells were observed under a laser confocal imaging analysis system, scale bar: 7 μm. **(E)** Statistical analysis of TRAIP distribution in cytoplasm and nucleus in HeLa cells was counted.

As shown previously, SUMO modification played a key role in its nuclear import (52, 53). We therefore examined the effect of overexpression of nsp1α on the nuclear distribution of TRAIP. The results showed that the distribution of TRAIP in the nucleus was significantly reduced, while that in the cytoplasmic was increased conversely when nsp1α was overexpressed (Figure 8A). Similarly, nsp1α can also affect the distribution of TRAIP K205R mutant (Figure 8B). Detection of endogenous TRAIP protein distribution further validated the result (Figure 8C). To visually detect the distribution of TRAIP in the cytoplasm and nucleus, the TRAIP immunofluorescence was analyzed at the indicated time points (Figure 8D). Statistics showed PRRSV nsp1α significantly reduced the TRAIP content in the nucleus (Figure 8E). Collectively, these results suggest nsp1α alters the distribution in the nucleus and cytoplasm and reduces the abundance of TRAIP in the nucleus.

### Nsp1α Expression Can Increase K48-Linked Ubiquitination of TBK1

**Figure 9:**
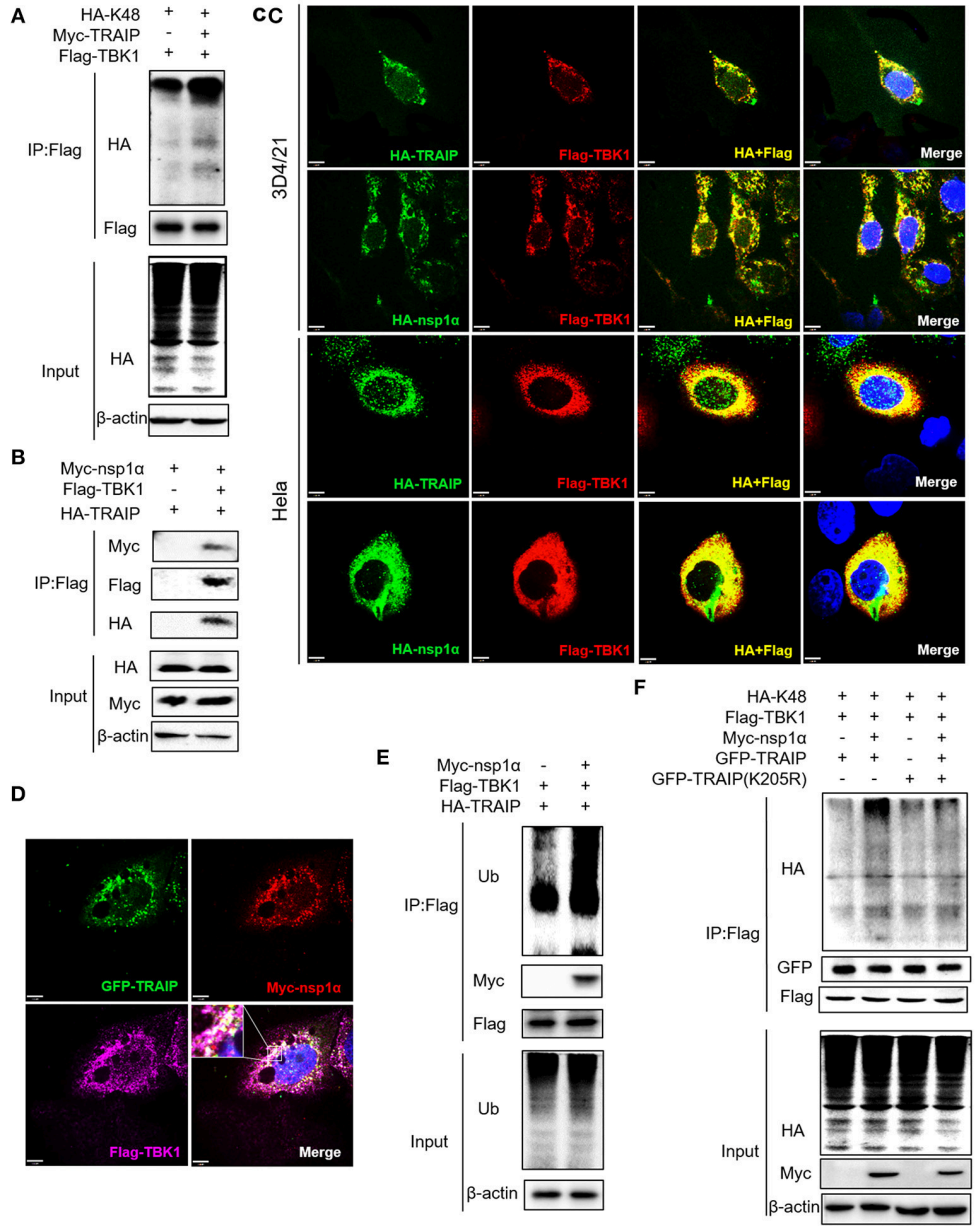
Nsp1α forms trimer complex with TRAIP and TBK1. **(A,F)** Detection of Lys48 (K48)-linked polyubiquitination of TBK1. HEK 293T cells were transfected with Flag-TBK1, Myc-TRAIP, HA-K48, or Myc-empty vector **(A)** or HA-K48, GFP-TRAIP or GFP-TRAIP (K205R), Flag-TBK1, Myc-nsp1α, or Myc-empty vector **(F)**. At 24 h post-transfection, FLAG-immunoprecipitates were probed with HA antibodies to detect to Lys48 (K48)-linked polyubiquitination of TBK1. **(B)** HEK293T cells were co-transfected with Myc-nsp1α, HA-TRAIP, Flag-TBK1, or Flag-empty vector. FLAG immunoprecipitation (IP) was used to detect the interaction of TBK1, nsp1α, and TRAIP. **(C)** Immunofluorescence was used to detect the interaction between TBK1, nsp1α, and TRAIP in 3D4/21 and HeLa cells. **(D)** Co-transfection of GFP-TRAIP with Myc-nsp1α and Flag-TBK1 into HeLa cells. The cells were fixed and double-stained with a mouse anti-Myc antibody and a rabbit anti-Flag antibody and followed by PE-conjugated anti-mouse IgG (red) and IF647 goat anti-rabbit IgG. Nuclei were stained with DAPI (blue). Cells were observed under a laser confocal imaging analysis system, scale bar: 7 μm. **(E)** HEK 293T cells were transfected with Flag-TBK1, HA-TRAIP, or Myc-empty vector. At 24 h post-transfection, FLAG-immunoprecipitates were probed with Ub antibodies to detect polyubiquitination of TBK1.

We have shown that nsp1α regulated the distribution of TRAIP in the cytoplasm and nucleus by removing both the SUMO modification and K48-ubiquitination modifications of TRAIP. Previous research has found that TRAIP promoted TBK1 degradation via K48-linked ubiquitination (39) and this result was verified in this report (Figure 9A). It was found Flag-TBK1 can interact with Myc-nsp1α and HA-TBK1 by immunoprecipitation (Figure 9B). The trimer complex of nsp1α with TRAIP and TBK1 was further confirmed in HeLa and 3D4/21 cells by immunofluorescence (Figures 9C,D). Furthermore, nsp1α promoted the K48-linked ubiquitination of TBK1 by TRAIP (Figures 9E,F). Therefore, it appeared that nsp1α increased TRAIP cytoplasmic abundance, leading to excessive TBK1 K48-linked ubiquitination.

### Nsp1α Promotes the Inhibitory Effect of TRAIP on Interferon-Mediated Innate Immunity

**Figure 10:**
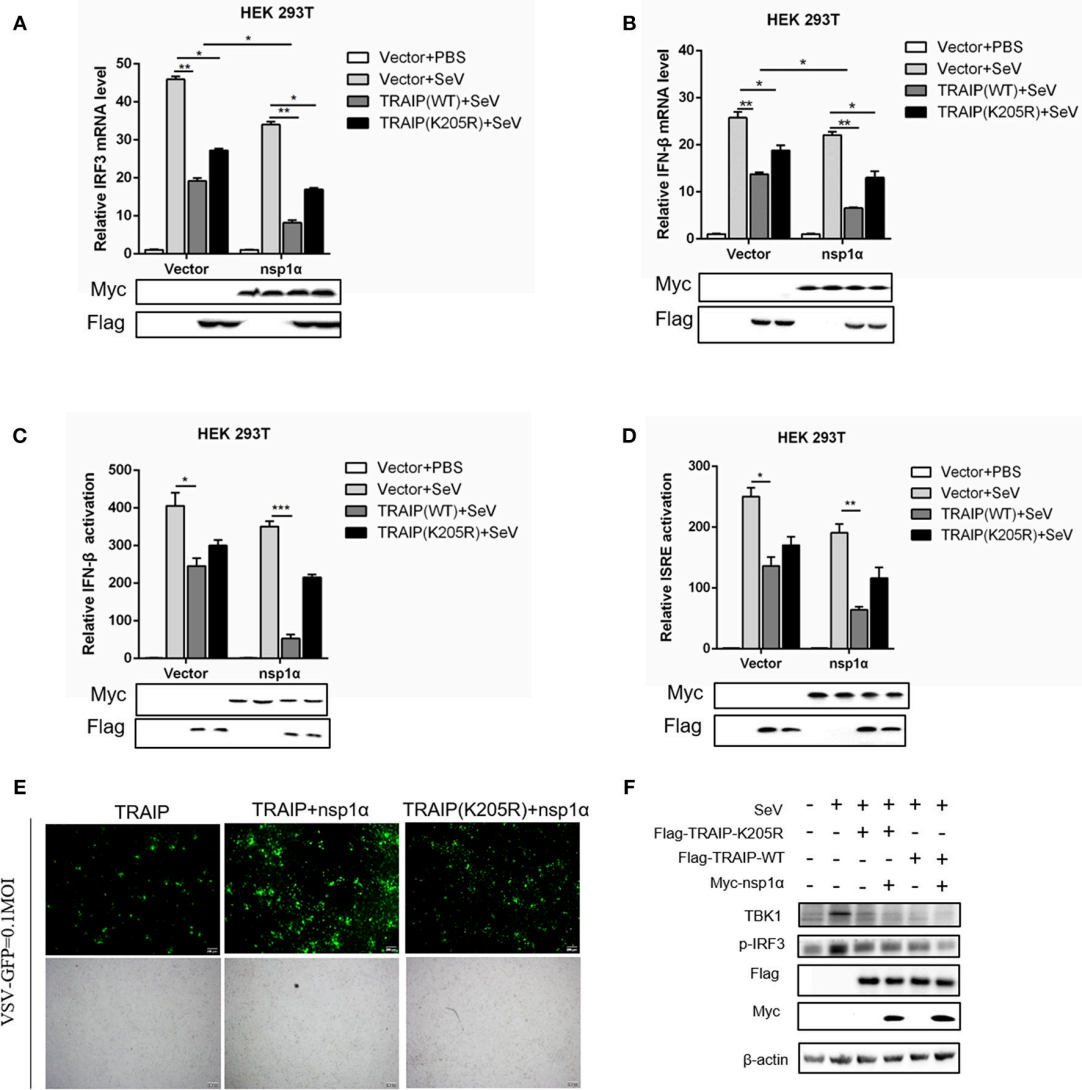
The inhibitory effect of TRAIP and nsp1α on interferon-mediated innate immunity. **(A,B)** HEK293T were transfected with TRAIP or TRAIP(K205R)together with Myc-nsp1α or Myc-empty vector respectively and then infected with 0.1 MOI SeV for 12 h. Cells were harvested, mRNA expression of IRF3 **(A)** and IFN-β **(B)** were analyzed by qRT-PCR, and TBK1 and p-IRF3 were detected by Western blotting **(F)**. **(C,D)** HEK293T were transfected with TRAIP or TRAIP(K205R)and Myc-nsp1α or Myc-empty vector together with IFN-β reporter or ISRE reporter constructs for 18h and then infected with 0.1 MOI SeV before being lysed for luciferase assays. **(E)** HEK293T were transfected with TRAIP or TRAIP(K205R)together with Myc-nsp1α or Myc-empty vector and then infected with 0.1 MOI VSV-GFP, immunofluorescence microscopy imaging detected the proliferation of VSV.

Type I (IFN-α and β) are parts of the non-specific immune system and serve as the first line of defense (1). Studies have found that TRAIP plays an important role in RIG-I-mediated type I interferon response. In our work, the function of TRAIP in the interferon signaling pathway induced by PRRSV was also examined. The results demonstrated that TRAIP significantly inhibited the production of type I interferons (IFN-α, IFN-β) and inflammatory related factors (IL-1β, IL-6, TNF-α) (Supplementary Figures 4a,b). While expression of TRAIP was downregulated by siRNA, the mRNA levels of IFN-α, IFN-β, and inflammatory cytokines (IL-1β, IL-6, TNF-α) increased (Supplementary Figures 4c–g). Then, the role of PRRSV nsp1α and TRAIP in interferon-mediated innate immunity was examined. Nsp1α significantly enhanced TRAIP inhibition of IRF3 and IFN-β mRNA levels in SeV-stimulated interferon activation (Figures 10A,B). Consistently, the luciferase reporter system further confirmed that TRAIP inhibited IFN production together with PRRSV nsp1α, and the downregulation of the IFN-β and ISRE activation was remarkable in cells co-transfected with Myc-nsp1α and Flag-TRAIP (Figures 10C,D). The vesicular stomatitis virus (VSV-GFP) was used to further substantiate the presence of biologically active IFN. As shown in Figure 10E, TRAIP promoted VSV proliferation while co-expression of nsp1α. Next, the expression of TBK1 and phosphorylated IRF3 in the interferon signaling pathway were examined by western blotting. The results showed nsp1α enhanced the effect of TRAIP on TBK1 degradation and IRF3 activation (Figure 10F). Consistently, the additive effect disappeared when the K205R mutation of TRAIP was present. Collectively, these data together reflected the biological activity of the interaction of nsp1α with TRAIP in the regulation of antiviral innate immune responses.

## Discussion

Post-translational modifications (PTMs) of proteins are critical for controlling essential cellular processes. Both ubiquitination and SUMOylation are among the most common post-translational modifications. The ubiquitin molecule contains seven lysine sites (K6, K11, K27, K29, K33, K48, and K63). Target proteins linked by the K48 ubiquitin chain can be recognized and degraded by the proteasome, and ubiquitin–protein ligases (E3s) play a crucial role in this process by recognizing target proteins. Ubiquitination changes the interaction between proteins, the stability and degradation of key proteins in signal pathways, thereby regulating natural immunity. SUMOylation is similar to the ubiquitination process, and reversibly modifies many proteins rather than perform proteasome-mediated degradation. SUMOylated proteins are more stable and SUMOylation modifications have extensive functions that are mainly reflected in their modified substrates. For example, TRAIP is a SUMO substrate and its activity is regulated by the SUMO machinery. SUMO-modified TRAIP has been reviewed in the regulation of protein localization and antagonism of ubiquitination. The SUMO modification of TRAIP guarantees its proper subcellular localization (46).

**Figure 11:**
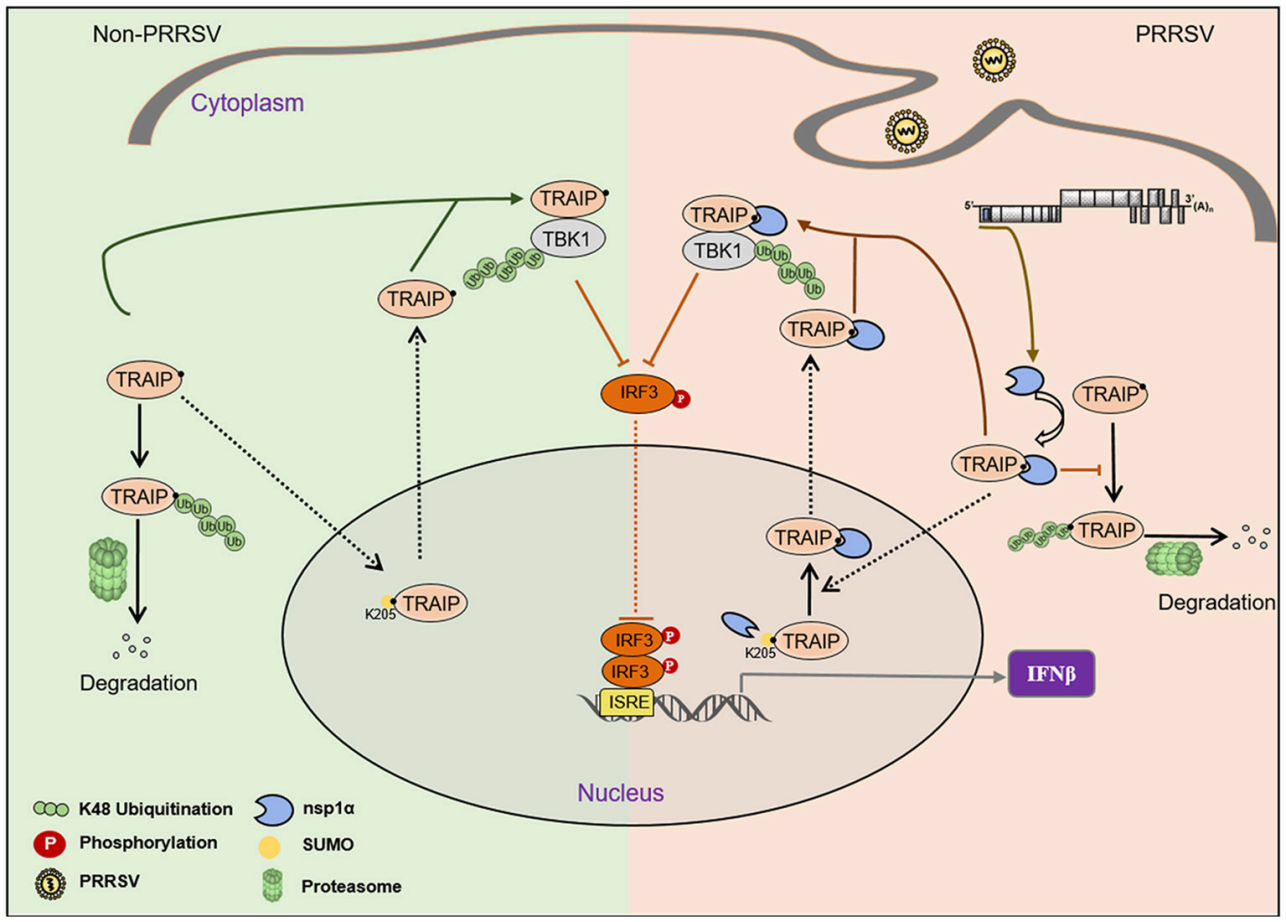
Proposed model for the regulation of the interaction of PRRSV nsp1α with post-translational SUMO modification of TRAIP. Compared to non-PRRSV infection model, PRRSV-nsp1α alters the role of TRAIP in cellular life activities by modulating post-translational modifications of proteins. (I) PRRSV infection inhibits SUMOylation and self-ubiquitination of TRAIP, resulting in excessive enrichment of TRAIP in the cytoplasm. (II) Enrichment of cytoplasmic TRAIP leads to excessive K48 ubiquitination and degradation of TBK1, resulting in reduced phosphorylated IRF3 and type I interferon production.

The virus employs multiple strategies to promote their own common proliferation in infected host cells. Viral proteins do not only participate in the regulation of the SUMOylation modification system, but also utilize the SUMOylation modification system to regulate other signaling pathways (54). In this study, the complete coding sequence (CDS) of TRAIP was cloned from porcine peripheral blood mononuclear cells (PBMC) and its amino acid sequence was highly homologous to *Homo sapiens*, including the RING domain and multiple SUMO sites. The SUMOylation and K48-linked polyubiquitination of TRAIP was attenuated by PRRSV nsp1α, thereby affecting intracellular localization of TRAIP and changing the distribution in the nucleus and cytoplasm. Consequently, these changes in turn affected the regulation of host immune signaling pathways. Our model for the regulation of the interaction of PRRSV nsp1α with post-translational modification of TRAIP is presented in Figure 11.

PRRSV is an important pathogen of swine. PRRSV-induced TRAIP transcription levels peak in early infection, which is consistent with the stage of high transcriptional expression levels of non-structural proteins during PRRSV replication and proliferation (55). Interestingly, we further observed that the morphology and subcellular localization of TRAIP was similar to nsp1α. What's more, PRRSV proliferation showed a trend of increasing in TRAIP overexpression in 3D4/21 cells.

There is crosstalk between SUMOylation and other post-translational modifications (56), and SUMOylation can compete with ubiquitination for substrate lysine residues to prevent proteasome degradation and ensure its stability (57, 58). However, some studies have shown that substrate proteins modified by SUMO as labeled molecules can recruit SUMO-targeted ubiquitin ligases (STUbL) to mediate subsequent ubiquitination degradation (50, 59, 60). Analysis revealed that most of SUMO-modified target molecules contained ψ-Lys-X-Glu (ψ representing an aliphatic amino acid, X being any amino acid) sites that specifically bind to SUMO (61). Subsequently, SUMO-linked TRAIP was verified and SUMO sites in TRAIP substrates have also been characterized (46). The LZ domain of TRAIP interacts with the PCPα domain of PRRSV nsp1α, and the K205 site in TRAIP was further confirmed as the key site. The specific mechanism of nsp1α control of TRAIP SUMO modification independent of the binding position. We hypothesized that nsp1α may affect the multi-step enzymatic process in SUMOylation or deSUMOylation. Interestingly, nsp1α not only inhibited SUMOylation but also reduced K48 ubiquitination of TRAIP. The K48 polyubiquitination of TRAIP was also inhibited by deleting the LZ domain of TRAIP, indicating that the ubiquitination sites in the LZ domain of TRAIP are crucial. Nsp1α may occupy or occlude the ubiquitination site in the LZ domain by changing the TRAIP spatial conformation, resulting in nsp1α inhibiting K48 ubiquitination of TRAIP and further stabilizing its structure.

Both nsp1α and TRAIP have been identified as nuclear shuttling proteins. The subcellular distribution of proteins is affected by the presence of nuclear localization signals (NLS) and nuclear export signals (NES) (62). Previous research has proposed that a nuclear export signal in PRRSV nsp1α is necessary for type I IFN inhibition (62), and PRRSV nsp1α may enter the cell nucleus through interaction with cellular proteins (63). Our study suggests that TRAIP containing an NLS is a potential cellular molecule assisting nsp1α into the nucleus, and TRAIP may also enter the cytoplasm as a partner of nsp1α. Nsp1α increased the cytoplasmic abundance and stability of TRAIP, further promoting TBK1 degradation via K48-linked ubiquitination in 293T cells. In addition, nsp1α, which appears to act as a partner molecule, forms a ternary complex with TRAIP and TBK1 in the cytoplasm. Ubiquitination modification not only regulates a number of physiological functions within the cell, but is also involved in the regulation of a variety of viral replication and proliferation processes. E3 ubiquitin ligases have been reported to be involved in the regulation of protein stability in RIG-I signaling (64–66).

Overall, our study elucidates a unique novel mechanism by which PRRSV nsp1α resists innate immunity and promotes virus proliferation by modulating TRAIP protein nuclear:cytoplasmic ratio. As showed in Figure 2, TRAIP was identified as a protein that is overexpressed by PRRSV infection and favors virus proliferation. Mechanistically, nsp1α inhibits SUMOylation and self-ubiquitination of TRAIP, inducing over-enrichment of TRAIP in the cytoplasm. As a nuclear transport protein, TRAIP has immune regulation functions for life activities of the cell. Enrichment of nsp1α-induced TRAIP cytoplasm also leads to excessive K48 ubiquitination and degradation of TBK1, thus impairing type I interferon production. This study proposes a new mechanism for PRRSV dual regulation modification of host proteins to affect innate immunity. However, whether nsp1α regulates TRAIP through other patterns besides steric hindrance, such as enzyme regulation, is still unknown. The effects of PRRSV on TRAIP post-translational modification or nuclear ratio in the context of virus-infected cells require more data to prove. And the mechanism of PRRSV infection induced excessive transcription and expression of TRAIP also demands further exploration.

## Author's Note

Tumor necrosis factor (TNF) receptor associated factors (TRAF) interacting protein (TRAIP) is a particular host protein that exerts multiple functions in cell cycle progression, DNA damage response, and DNA repair pathways. Currently, the mechanism of action of TRAIP in PRRSV infection has never been reported. In this study, the relationship between TRAIP and porcine reproductive and respiratory syndrome virus replication (PRRSV) was investigated. Small ubiquitin-like modifier self-addition (SUMOylation) and self-ubiquitination of TRAIP was attenuated by PRRSV non-structural protein 1α (nsp1α), thereby affecting intracellular localization of TRAIP and changing the distribution in the nucleus and cytoplasm. As a cytoplasmic event, the cytoplasmic guiding effect of nsp1α on TRAIP promotes the ubiquitination and degradation of serine/threonine-protein kinase (TBK1). To sum up, a novel mechanism was presented by which PRRSV utilizes host proteins to regulate innate immunity. This study enriches the understanding of viral regulatory host protein post-translational modifications and interference with cell life processes.

## Author Contributions

JH conceived and designed the experiments. PS, YS, RL, CC, Liz, and LeZ performed the experiments. YS, PS, and KF analyzed the data. JH contributed reagents, materials, analysis tools. PS and JH wrote the paper.

### Conflict of Interest Statement

The authors declare that the research was conducted in the absence of any commercial or financial relationships that could be construed as a potential conflict of interest.
