# Supplementary material for: Dual Regulation of Host TRAIP Post-translation and Nuclear/Plasma Distribution by Porcine Reproductive and Respiratory Syndrome Virus Non-structural Protein 1α Promotes Viral Proliferation
Source: Front Immunol. 2018 Dec 18;9:3023. doi: 10.3389/fimmu.2018.03023 (PMC6305329; doi:10.3389/fimmu.2018.03023)
Supplement: Supplementary file 1 [file Data_Sheet_1.PDF]

Supplementary material

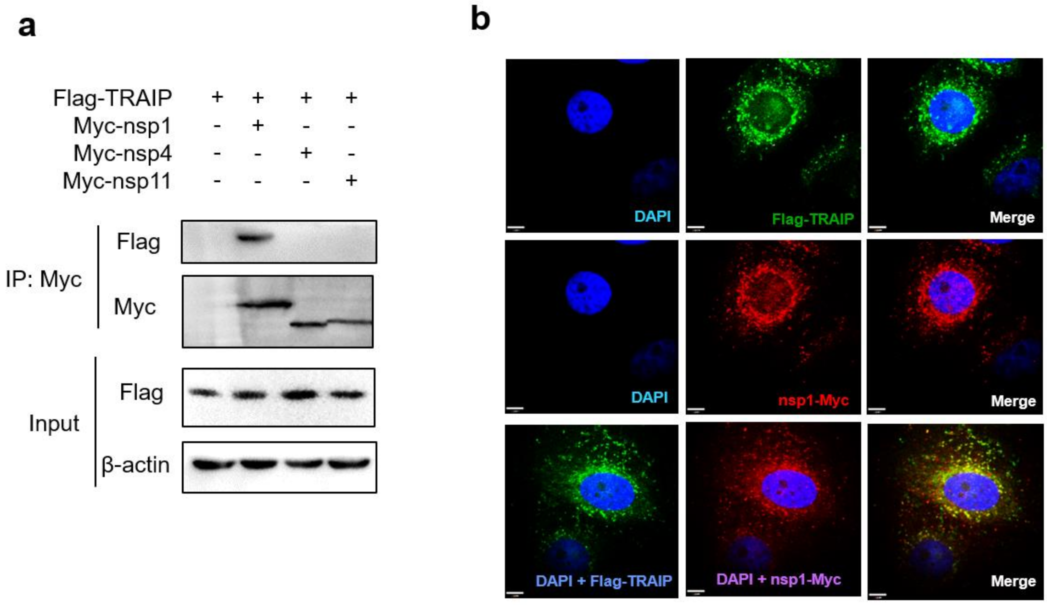

Supplementary Fig.1. TRAIP interacts with PRRSV-Nsp1. HEK293 cells were co-transfected with Flag-TRAIP plasmid and Myc-nsp1 or Myc-nsp4 or Myc-nsp11 or empty vector plasmid, respectively. 24 h after transfection, the cell lysates were co-immunoprecipitated with an anti-Myc mAb and probed with anti-Flag mAb and anti-Myc antibody respectively by Western blotting. (b) Co-localization of nsp1 protein with TRAIP in Hela cells. Hela cells were co-transfected with Flag-TRAIP, Myc-nsp1 plasmid, respectively. The cells were fixed at 18h post-transfection and processed by immunostaining with double-stained with a rabbit anti-Myc mAb and a mouse anti-Flag antibody and followed by FITC-conjugated anti-mouse IgG (green) and PE-conjugated anti-rabbit IgG (red). Nuclei were stained with DAPI. Cells were observed under a laser confocal imaging analysis system, scale bar: 7 μm.

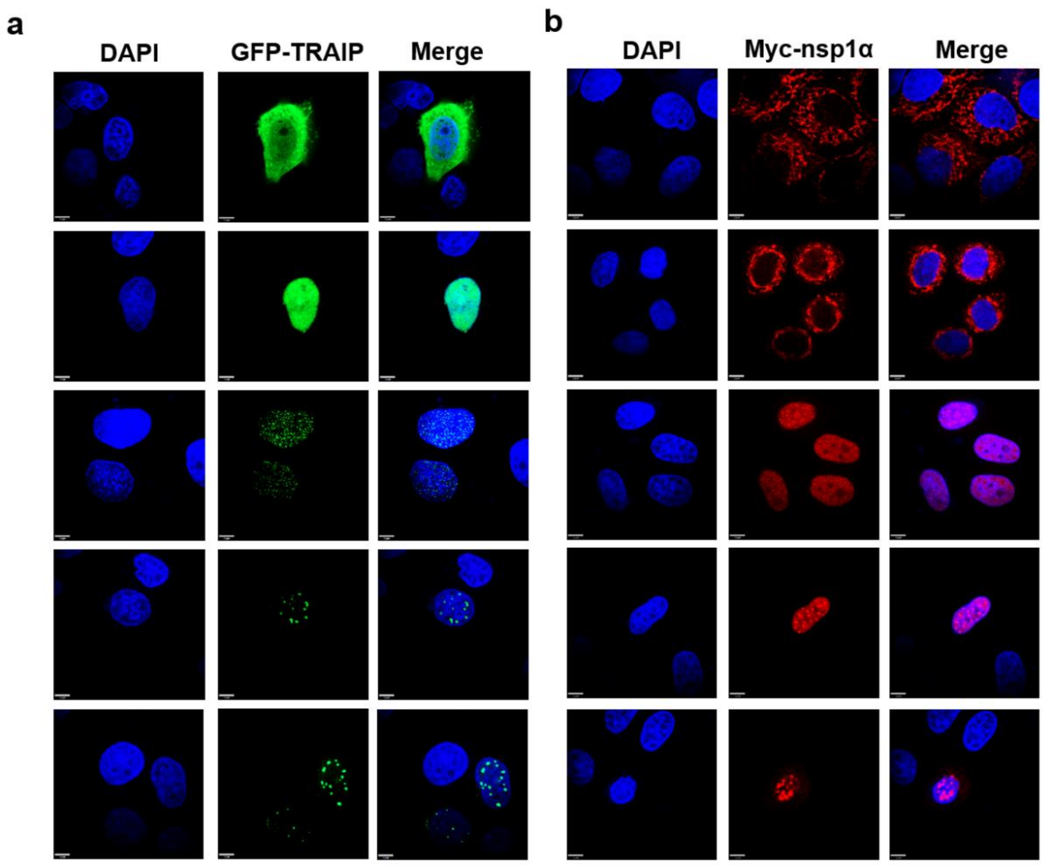

Supplementary Fig.2. Subcellular localization of TRAIP and nsp1α HeLa cells were seeded in 12-well plates and transfected with GFP-TRAIP expressing plasmid (a) or transfected with Myc-nsp1α expressing plasmid (b). At 24h post-transfection, the cells were fixed and permeabilized with Triton X-100. Cells were then processed by immunostaining directly (a) and incubated a rabbit anti-Myc mAb for 1h and followed by PE-conjugated anti-rabbit IgG (red). Nuclei were stained with DAPI. Cells in different fields of view were observed under a laser confocal imaging analysis system, scale bar: 7 μm.

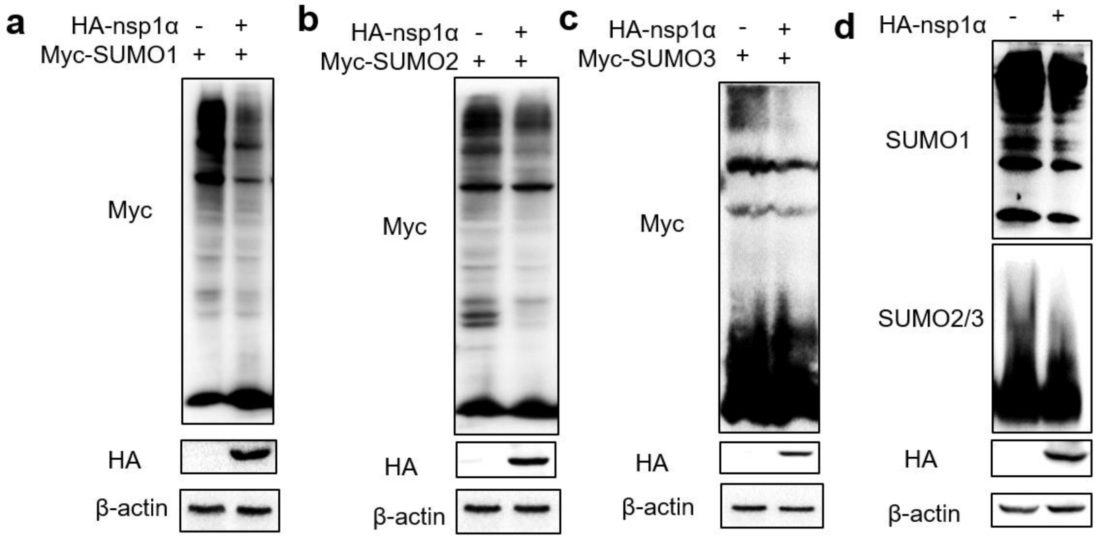

Supplementary Fig.3. Nsp1α can remove the SUMO modification of cells. HEK 293T cells were co-transfected with HA-nsp1α and Myc-SUMO1 (a), Myc-SUMO2 (b) or Myc-SUMO3(c), at 24h post-transfected, effect of nsp1α on exogenous SUMO modification were detected by WB. (d) 293T cells were transfected with HA-nsp1α or vector, at 24h post-transfected, effect of nsp1α on endogenous SUMO modification were detected by WB.

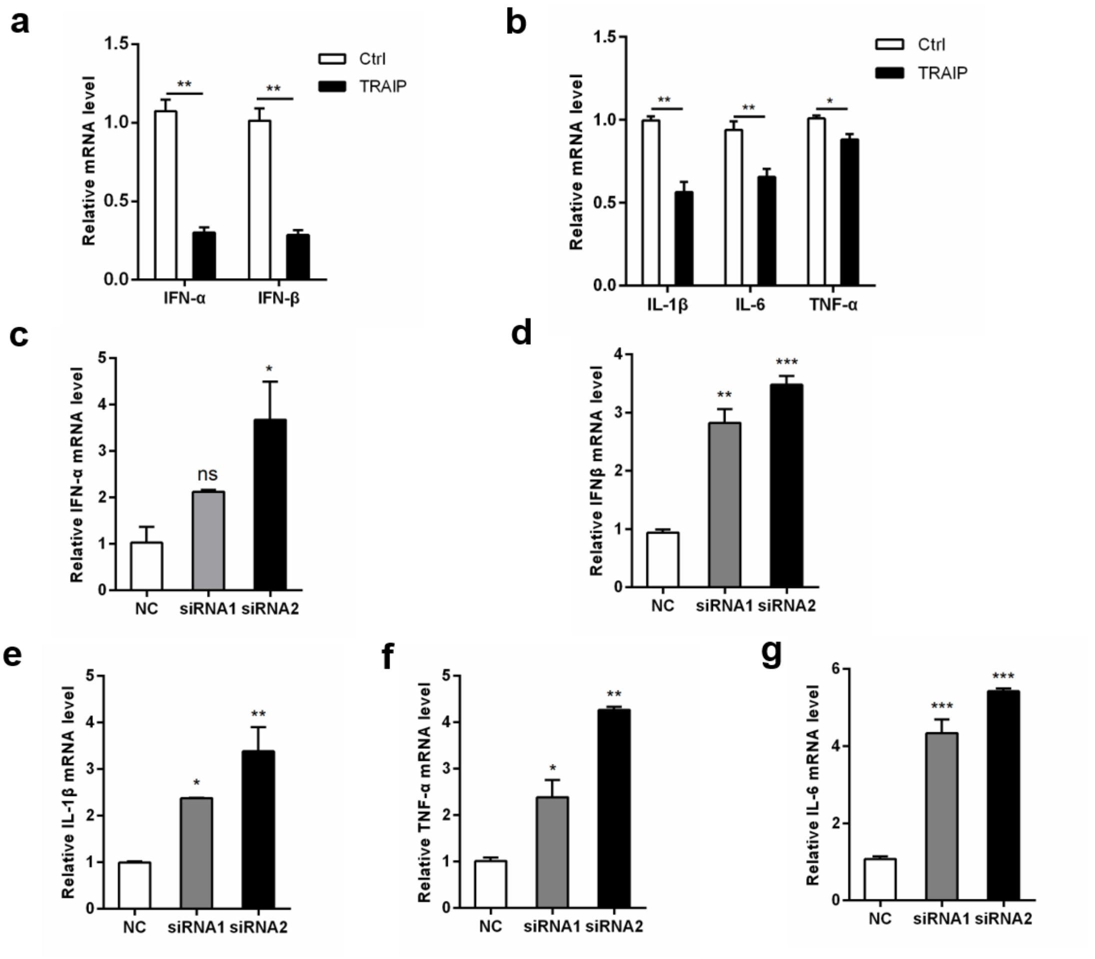

Supplementary Fig.4. Effects of TRAIP on interferon signal pathway induced by PRRSV. 3D4/21 cells were transfected with siRNA1, siRNA2 and Flag-TRAIP respectively. At 12h post-transfected, the cells were infected with PRRSV at a multiplicity of infection (MOI) of 0.5. RNA was harvested at the indicated time post-treatment and the relative expression of Type I interferon signaling factor for qRT-PCR analysis. The expression of IFN-α and IFN-β (a), IL-1β, IL-6 and TNF-α (b) overexpress TRAIP. The expression of IFN-α (c), IFN-β (d), IL-1β (e), IL-6(f) and TNF-α (g) was analyzed by real time RT-PCR transfected siRNA1, siRNA2 in 3D4/21 cells. *P < 0.05**P<0.01; ***P<0.001 (analysis of two-way ANOVA followed by Bonferroni post-test). Data are representative of three independent experiments.
